# Dimeric Labdane Diterpenes: Synthesis and Antiproliferative Effects

**DOI:** 10.3390/molecules18055936

**Published:** 2013-05-21

**Authors:** Mariano Walter Pertino, Cristina Theoduloz, Marco Bastías, Guillermo Schmeda-Hirschmann

**Affiliations:** 1 Instituto de Química de Recursos Naturales, Universidad de Talca, Casilla 747, Talca, Chile; 2 Facultad de Ciencias de la Salud, Universidad de Talca, Talca, Chile

**Keywords:** labdane diterpene dimers, click chemistry, semisynthesis, antiproliferative activity

## Abstract

Several diterpenes with the labdane skeleton show biological activity, including antiproliferative effects. Most of the research work on bioactive labdanes has been carried out on naturally occurring diterpenes and semisynthetic derivatives, but much less is known on the effects of diterpene dimers. The aim of the present work was to synthesize dimeric diterpenes from the labdane imbricatolic acid using esters, ethers and the triazole ring as linkers. Some 18 new derivatives were prepared and the compounds were evaluated for antiproliferative activity on human normal fibroblasts (MRC-5) and the following human tumor cell lines: AGS, SK-MES-1, J82 and HL-60. The diethers **8**–**10**, differing in the number of CH_2_ units in the linker, presented better antiproliferative activity with a maximum effect for the derivative **9**. The best antiproliferative effect against HL-60 cells was found for compounds **3** and **17**, with IC_50_ values of 22.3 and 23.2 µM, lower than that found for the reference compound etoposide (2.23 µM). The compounds **9**, **17** and **11** were the most active derivatives towards AGS cells with IC_50_ values of 17.8, 23.4 and 26.1 µM. A free carboxylic acid function seems relevant for the effect as several of the compounds showed less antiproliferative effect after methylation.

## 1. Introduction

Natural product monomers occur in all living organisms and many can also form polymeric structures, including rubber, cellulose or lignin. Less common are dimeric compounds formed by coupling two units by C-C, ester, ether, C-N or N-N bonds. 

It has been reported that dimeric compounds can be employed as potential anticancer agents because they might interact with two different binding sites on a receptor or on two separate monomers of a dimeric protein [1]. In some cases, it has been observed that dimers not only maintained the activity shown by its monomer, but could also increase it. The dimer montamine from *Centaurea montana* presented two times higher activity on *in vitro* colon cancer than its monomer [2]. Monomeric ergolines presented weak antiplasmodial effect, while dimerized ergoline derivatives, prepared using different aliphatic or arylalkyl spacers, showed significantly increased activity [3].

Naturally occurring dimers also show antiproliferative activity. A few examples include benzoquinone derivatives [4], flavones [5], alkaloids [6], triterpene saponins [7] and diterpenes [8], among others. Dimeric terpenoids have a much restricted distribution in Nature and several of them display relevant biological effects such as antiprotozoal [9], antioxidant and cytotoxic [7], apoptotic [8] or TRAIL-resistance overcoming activity [10]. The dimeric diterpenes described so far belong to several chemical skeletons, including icetexanes [11], cassaines [10], abietanes [12], kauranes [13,14] and labdanes [15].

Different biological activities have been reported for labdanes [15,16,17,18,19,20] including antiproliferative effects [21]. Several labdane derivatives were prepared and assessed for gastroprotective and cytotoxic effects looking for structure-activity relationships. The semisynthetic labdanes included esters, ethers, amides with aromatic amines [17], amino acids [22], as well as hybrid molecules formed with naphthoquinones [23]. This paper describes the synthesis of some new dimeric diterpenes including esters, ethers and dimers fused by triazole rings, starting from the naturally occurring labdane imbricatolic acid. The new compounds were evaluated for antiproliferative activity on human normal lung fibroblasts and selected cancer cell lines. 

Other studies on the synthesis and cell toxicity of dimeric compounds include the work on dimeric epothilone A derivatives, prepared by using diacyl spacers. The new compounds were evaluated on tubulin polymerization and the cytotoxicity was determined against cancer cell lines [24]. All the dimers were less cytotoxic than epothilone, however, several dimeric compounds inhibit endothelial cell differentiation and endothelial cell migration. Looking for new compounds with antimalarial and anticancer activity, C-10 non-acetal dimers of 10β-(2-hydroxyethyl)deoxoartemisinin were prepared and evaluated on cell lines [25]. All the artemisinin dimers showed potent antimalarial activity in the nM range. One of the dimers evaluated *in vitro* was fifty times more potent than artemisinin. The cytotoxicity of the new products was determined on the National Cancer Institute (NCI) tumour cell panel, most of the compounds, except the phosphate dimer were not cytotoxic. The synthesis, cytotoxicity, *in vivo* anticancer and antiprotozoal effect of twelve artemisinin acetal dimers was reported [26]. Several of the new dimers were more active than the corresponding monomers on the cancer cell lines tested with GI_50_ values for the dimers between 8.7 and 0.019 μM. Hybrid compounds combining the artemisinin and a quinoline moiety were synthesized [27] and evaluated against *Plasmodium falciparum*. Two of the new compounds showed excellent activity against the protozoa* in vitro*.

## 2. Results and Discussion

**Scheme 1:**
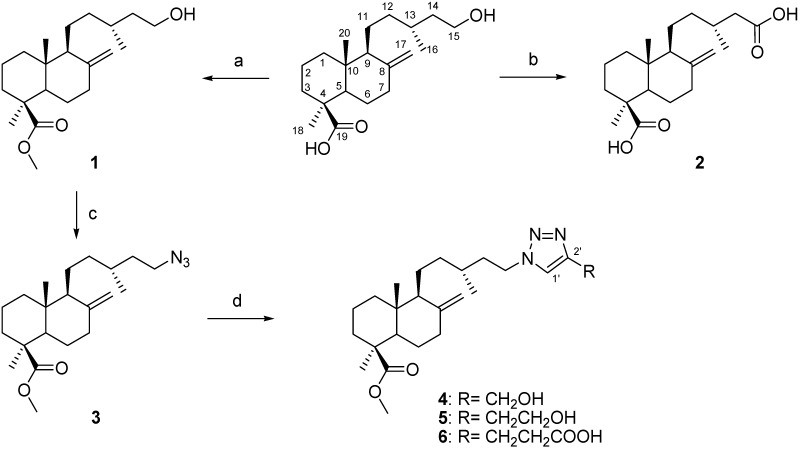
Preparation of derivatives **1–6** from imbricatolic acid.

Starting from the naturally occurring diterpene imbricatolic acid, fourteen dimers were synthesized using different linkers. The diterpene imbricatolic acid, used as starting compound for the synthesis was isolated from the resin of *Araucaria araucana.* Imbricatolic acid was methylated with diazomethane to form compound **1**. 15-Hydroxyimbricatolic acid was treated with the Jones reagent (CrO_3_/H_2_SO_4_/H_2_O) to afford the diacid junicedric acid (**JA**, **2**). Compound **1** was tosylated and then treated with NaN_3_ in DMF to form the azide **3**. Compounds **4–6** were prepared by “click chemistry” of **3** with different alkynes using CuSO_4_·5H_2_O/sodium ascorbate in* t*-BuOH/H_2_O (Scheme 1). 

*Reagents and conditions*: (a) CH_2_N_2_, Et_2_O, 91%; (b) Jones oxidation: CrO_3_, H_2_SO_4_, H_2_O, acetone, 84%; (c) (i) TsCl, pyridine 78%; (ii) NaN_3_, DMF, 84%; (d) appropriate alkyne, CuSO_4_·5H_2_O, sodium ascorbate, *t*-BuOH/H_2_O 1:1, 68%–73%.

**Scheme 2:**
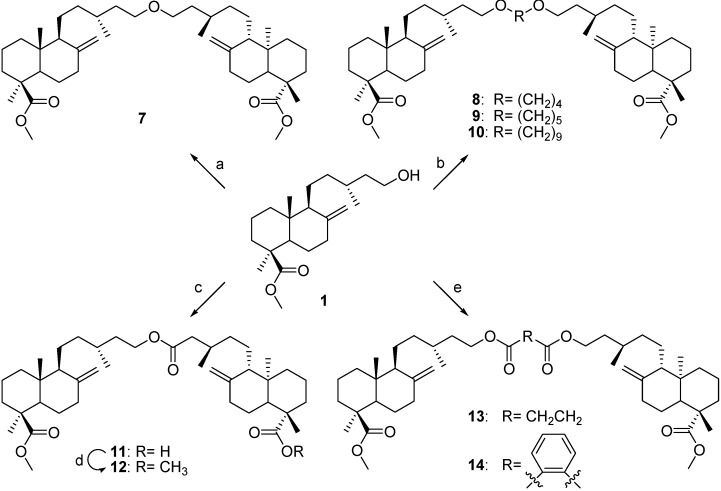
Preparation of derivatives **7#x2013;14** from imbricatolic acid methyl ester (**1**).

To prepare the ether 7, compound **1** was treated with NaH in DMF under constant stirring and then the tosylated compound **1** was added to the solution. The ethers **8–10** were prepared from **1** with NaH in DMF and the corresponding dibromoalkanes. To prepare the ester **11**, the diterpene **JA (2)** dissolved in dry CH_2_Cl_2_ was treated with *N*,*N'*-dicyclohexylcarbodiimide (DCC)/dimethylaminopyridine (DMAP) and compound **1**. Compounds **13** and **14** were prepared from compound **1** with DCC/DMAP in dry CH_2_Cl_2_ and succinic acid and phthalic acid, respectively (Scheme 2).

*Reagents and conditions*: (**a**) (i) TsCl, Pyridine, 78%; (ii) compound **1,** NaH, DMF, 51%; (**b**) appropriate dibromoalkane, NaH, DMF, 41%–56%; (**c**) compound 2, DCC, DMAP, CH_2_Cl_2_, 65%; (**d**) CH_2_N_2_, Et_2_O, 92%; (**e**) appropriate diacid, DCC, DMAP, CH_2_Cl_2_, 45%–53%.

**Scheme 3:**
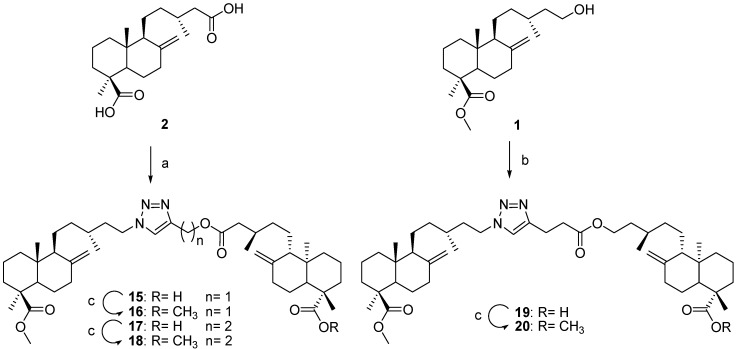
Preparation of derivatives **15–20** from **1** and **2** by click chemistry.

*Reagents and conditions*: (**a**) (i) appropriate alkyne alcohol, DCC, DMAP, CH_2_Cl_2_, 68%–73%; (ii) compound **3**, CuSO_4_.5H_2_O, sodium ascorbate, *t*-BuOH/H_2_O 1:1, 66%–74%; (**b**) (i) 4-pentynoic acid, DCC, DMAP, CH_2_Cl_2_, 71%; (ii) compound **3**, CuSO_4_·5H_2_O, sodium ascorbate, *t*-BuOH/H_2_O 1:1, 62%; (**c**) CH_2_N_2_, Et_2_O, 86%–94%.

The dimers **15**, **17** and **19** were prepared by “click chemistry”. To prepare the dimers **15** and **17**, JA (**2**) was treated with DCC/DMAP in dry CH_2_Cl_2_ and different alkyne alcohols (propargyl alcohol and 3-butyn-1-ol), to afford the corresponding alkynes esters. These esters were treated whit the azide **3** and CuSO_4_·5H_2_O/sodium ascorbate in* t*-BuOH/H_2_O to yield the desired products. In the same way **19** was obtained treated imbricatolic acid with DCC/DMAP in dry CH_2_Cl_2_ and 4-pentynoic acid, the ester obtained was then treated with the azide 3 (Scheme 3). All the C-19 methyl esters were prepared by reaction with a diazomethane solution (compounds **12**, **16**, **18** and **20**). In all, some 19 compounds were prepared starting from the diterpenes labd-8(17)-en-15-hydroxy-19-oic acid (imbricatolic acid) and labd-8(17)-en-15,19-dioic acid (junicedric acid, compound **2**). The new compounds include ethers and esters with different “linkers” (spacers) as well as 1,2,3-triazole-substituted derivatives prepared by click chemistry. 

The purity of all derivatives was over 98% as assessed by ^1^H-NMR spectroscopy. The syntheses are summarized in Scheme 1, Scheme 2, Scheme 3. All of the compounds were characterized by spectroscopic means and compounds **3–20** are described for the first time. The dimeric compounds prepared can be classified into symmetric dimers (compounds **7–10**, **13–14**) and asymmetric dimers (compounds **11–12**, **15–20**). Unlike the symmetric dimers, asymmetric dimers showed small variations in many of their ^13^C-NMR signals (see Table 1 and Table 2). 1D and 2D-NMR methods were used for a better H and C assignation. Selected NMR spectra are presented as Supporting Information.

**Table 1:** ^13^C-NMR Data of Dimeric Symmetric Compounds **7–10**, **13–14**.

C	7	8	9	10	13	14
2 ×C-1	39.12 t	39.13 t	39.18 t	39.17 t	39.18 t	39.17 t
2 × C-2	20.03 t	20.01 t	19.99 t	19.98 t	19.99 t	19.99 t
2 × C-3	38.23 t	38.23 t	38.30 t	38.29 t	38.28 t	38.25 t
2 × C-4	44.32 s	44.32 s	44.31 s	44.29 s	44.32 s	44.42 s
2 × C-5	56.36 d	56.36 d	56.41 d	56.38 d	56.37 d	56.40 d
2 × C-6	26.28 t	26.28 t	26.26 t	26.27 t	26.28 t	26.32 t
2 × C-7	38.82 t	38.82 t	38.81 t	38.82 t	38.80 t	38.82 t
2 × C-8	148.39 s	148.41 s	148.38 s	148.34 s	148.32 s	148.35 s
2 × C-9	56.65 d	56.64 d	56.66 d	56.60 d	56.57 d	56.55 d
2 × C-10	40.37 s	40.37 s	40.35 s	40.35 s	40.37 s	40.40 s
2 × C-11	21.09 t	21.16 t	21.16 t	21.15 t	21.05 t	21.05 t
2 × C-12	36.43 t	36.43 t	36.44 t	36.44 t	36.09 t	36.14 t
2 × C-13	30.69 d	30.70 d	30.69 d	30.67 d	30.54 d	30.61 d
2 × C-14	36.33 t	36.30 t	36.34 t	36.34 t	35.16 t	35.02 t
2 × C-15	69.31 t	69.32 t	69.30t	69.23 t	63.51 t	63.57 t
2 × C16	19.91 q	19.88 q	19.92 q	19.82 q	19.69 q	19.69 q
2 × C-17	106.35 t	106.31 t	106.28 t	106.34 t	106.33 t	106.38 t
2 × C-18	28.79 q	28.79 q	28.82 q	28.85 q	28.86 q	28.85 q
2 × C-19	177.86 s	177.84 s	177.76 s	177.80 s	177.88 s	178.37 s
2 × C-20	12.54 q	12.55 q	12.55 q	12.56 q	12.58 q	12.58 q
2 × C-OMe	51.13 q	51.12 q	51.08 q	51.14 q	51.20 q	51.27 q

**R**: **8**: 69.81 (CH_2_, 2 × C-1''), 29.77 (CH_2_, 2 × C-2''); **9**: 70.54 (CH_2_, 2 × C-1''), 32.63 (CH_2_, 2 × C-2''), 24.96 (CH_2_, C-3''); **10**: 70.01 (CH_2_, 2 × C-1''), 32.82 (CH_2_, 2 × C-2''), 26.18 (CH_2_, 2 × C-3''), 29.77 (CH_2_, 2 × C-4''), 29.39 (CH_2_, C-5''); **13**: 172.29 (CO, 2 × C-1''), 28.94 (2 × CH_2_, C-2''); **14**: 168.84 (CO, 2 × C-1''), 131.29 (C,2 × C-2''), 129.39 (CH, 2 × C-3''), 130.86 (CH, 2 × C-4'').

**Table 2:** ^13^C-NMR Data of Dimeric Asymmetric Compounds **11–12**, **15–20**.

C	11	12	15	16	17	18	19	20
1	39.20 t	39.14 t	39.11 t *	39.17 t	39.18 t	39.20 t	39.19 t	39.11 t
1'	39.20 t	39.14 t	39.16 t *	39.17 t	39.18 t	39.20 t	39.19 t	39.11 t
2	20.05 t	19.94 t	19.94 t	19.95 t	19.96 t	20.00 t	19.96 t	19.91 t
2'	20.05 t	19.94 t	19.94 t	19.95 t	19.96 t	20.00 t	19.96 t	19.91 t
3	38.30 t	38.23 t	37.97 t *	37.12 t *	38.04 t *	37.24 t *	38.02 t *	37.11 t *
3'	38.30 t	38.23 t	38.20 t *	38.23 t *	38.24 t *	38.28 t *	38.25 t *	38.18 t *
4	44.32 s *	44.26 s	44.27 s *	44.28 s	44.31 s *	44.33 s	44.30 s *	44.28 s
4'	44.16 s *	44.26 s	44.13 s *	44.28 s	44.15 s *	44.33 s	44.16 s *	44.28 s
5	56.48 d *	56.31 d	56.32 d *	56.31 d *	56.35 d *	56.37 d *	56.35 d	56.29 d
5'	57.41 d *	56.31 d	56.41 d *	56.39 d *	56.42 d *	56.47 d *	56.35 d	56.29 d
6	26.27 t *	26.22 t	26.03 t *	26.23 t	26.07 t *	26.28 t	26.06 t *	26.21 t
6'	26.07 t *	26.22 t	26.23 t *	26.23 t	26.25 t *	26.28 t	26.26 t *	26.21 t
7	38.81 t	38.74 t	38.74 t	38.75 t	38.76 t	38.80 t	38.77 t	38.72 t
7'	38.81 t	38.74 t	38.74 t	38.75 t	38.76 t	38.80 t	38.77 t	38.72 t
8	148.25 s *	148.26 s*	148.19 s *	148.25 s *	148.25 s *	148.32 s *	148.26 s *	148.27 s *
8'	148.07 s *	148.13 s*	148.08 s *	148.17 s *	148.15 s *	148.23 s *	148.18 s *	148.23 s *
9	56.60 d	56.51 d	56.52 d	56.53 d	56.58 d	56.60 d	56.56 d	56.49 d
9'	56.60 d	56.51 d	56.52 d	56.53 d	56.58 d	56.60 d	56.56 d	56.49 d
10	40.36 s *	40.31 s	40.34 s *	40.34 s	40.37 s *	40.38 s	40.37 s *	40.31 s
10'	40.54 s *	40.31 s	40.51 s *	40.34 s	40.54 s *	40.38 s	40.56 s *	40.31 s
11	21.06 t *	21.00 t	21.11 t	21.13 t	21.18 t	21.22 t	21.01 t	21.01 t
11'	21.13 t *	21.10 t	21.11 t	21.13 t	21.18 t	21.22 t	21.01 t	21.01 t
12	36.10 t *	36.04 t *	35.85 t	35.87 t	35.91 t	35.96 t	36.09 t	36.05 t
12'	35.92 t *	35.89 t *	35.85 t	35.87 t	35.91 t	35.96 t	36.09 t	36.05 t
13	30.59 d *	30.53 d *	31.00 d	31.02 d	31.05 d	31.09 d	31.08 d	31.04 d
13'	31.07 d *	31.04 d *	31.00 d	31.02 d	31.05 d	31.09 d	31.08 d	31.04 d
14	35.32 t	35.25 t	35.77 t	35.78 t	35.82 t	35.85 t	35.83 t *	35.76 t *
14'	41.70 t	41.67 t	41.35 t	41.37 t	41.54 t	41.57 t	35.22 t *	35.14 t *
15	62.77 t	62.74 t	48.61 t	48.62 t	48.53 t	48.50 t	48.45 t	48.48 t
15'	173.40 s	173.40 s	173.18 s	173.20 s	173.25 s	173.19 s	63.17 t	63.23 t
16	19.75 q	19.78 q *	19.86 q *	19.87 q *	19.91 q *	19.97 q *	19.68 q *	19.61 q *
16'	19.75 q	19.69 q *	19.43 q *	19.44 q *	19.47 q *	19.51 q *	19.49 q *	19.40 q *
17	106.34 t *	106.34 t *	106.27 t *	106.26 t	106.28 t *	106.30 t *	106.30 t *	106.25 t
17'	106.44 t *	106.29 t *	106.34 t *	106.26 t	106.36 t *	106.36 t *	106.38 t *	106.25 t
18	28.83 q *	28.80 q	28.80 q *	28.81 q	28.82 q *	28.86 q	28.84 q	28.78 q
18'	29.04 q *	28.80 q	29.01 q *	28.81 q	29.05 q *	28.86 q	28.84 q	28.78 q
19	177.74 s	177.77 s	177.70 s	177.74 s;	177.81 s	177.78 s	177.95 s	178.00 s *
19'	183.27 s	177.77 s	183.14 s	177.74 s	182.74 s	177.78 s	182.74 s	178.03 s *
20	12.58 q *	12.53 q	12.55 q *	12.54 q;	12.58 q *	12.60 q	12.58 q *	12.52 q
20'	12.76 q *	12.53 q	12.73 q *	12.54 q	12.79 q *	12.60 q	12.79 q *	12.52 q
OMe	51.10 q	51.11 q	51.11 q	51.13 q	51.16 q	51.17 q	51.17 q	51.17 q
OMe'	-	51.11 q	-	51.13 q	-	51.17 q	-	51.17 q

**R**: **15**: 123.60 (CH, C-1''), 142.75 (C, C-2''), 57.36 (CH_2_, C-3''); **16**: 123.55 (CH, C-1''), 142.75 (C, C-2''), 57.40 (CH_2_, C-3''); **17**: 121.30 (CH, C-1''), 144.03 (C, C-2''), 21.02 (CH_2_, C-3''), 63.00 (CH_2_, C-4''); **18**: 121.16 (CH, C-1''), 144.05 (C, C-2''), 21.05 (CH_2_, C-3''), 63.03 (CH_2_, C-4''); **19**: 121.01 (CH, C-1''), 146.33 (C, C-2''), 29.05 (CH_2_, C-3''), 33.75 (CH_2_, C-4''), 172.92 (COO, C-5''); **20**: 121.17 (CH, C-1''), 146.27 (C, C-2''), 30.51 (CH_2_, C-3''), 33.78 (CH_2_, C-4''), 172.98 (COO, C-5''). * For dimeric compounds, pair of signals belonging to the same carbon in the different moieties are interchangeable.

The compounds **1–20** were assessed for antiproliferative effect towards human lung fibroblasts (MRC-5) and the following human tumor cell lines: gastric adenocarcinoma (AGS), lung cancer cells (SK-MES-1), bladder carcinoma (J82) and promyelocytic leukemia (HL-60). IC_50_ values >100 *µ*M were considered as inactive. The results are summarized in Table 3.

**Table 3:** Antiproliferative activity of compounds **1–20** against MRC-5 normal fibroblasts and selected tumor cell lines. *^a^*

Compound	(IC_50_ ± SD, *µ*M) *^b^*				
	MRC-5	AGS	SK-MES-1	J82	HL-60
**1**	77.7 ± 4.0	38.5 ± 1.6	59.7 ± 2.4	63.7 ± 2.6	47.5 ± 3.9
**2**	>100	>100	>100	>100	>100
**3**	91.8 ± 6.4	73.8 ± 5.2	67.6 ± 3.4	>100	22.3 ± 3.1
**4**	77.4 ± 3.9	41.5 ± 2.1	67.7 ± 3.1	60.7 ± 4.6	>100
**5**	75.8 ± 5.3	54.8 ± 3.3	57.2 ± 3.4	56.2 ± 3.9	48.9 ± 4.5
**6**	>100	>100	>100	>100	>100
**7**	>100	>100	>100	>100	>100
**8**	>100	70.3 ± 6.3	>100	>100	57.8 ± 4.1
**9**	68.1 ± 3.4	17.8 ± 1.3	67.5 ± 6.9	>100	32.6 ± 2.3
**10**	>100	>100	67.9 ± 5.4	>100	>100
**11**	71.2 ± 5.3	26.1 ± 2.2	>100	>100	80.3 ± 5.9
**12**	>100	>100	38.5 ± 2.8	61.4 ± 3.1	>100
**13**	76.8 ± 5.4	62.9 ± 3.9	68.2 ± 4.5	72.1 ± 4.3	60.8 ± 4.2
**14**	76.3 ± 5.8	51.7 ± 2.8	>100	>100	55.6 ± 3.9
**15**	>100	39.4 ± 2.4	56.0 ± 4.5	84.7 ± 6.8	44.1 ± 4.1
**16**	>100	>100	>100	>100	>100
**17**	79.6 ± 5.1	23.4 ± 1.4	67.5 ± 4.9	34.6 ± 2.5	23.2 ± 3.1
**18**	>100	>100	>100	>100	>100
**19**	>100	>100	>100	>100	42.4 ± 2.5
**20**	>100	>100	>100	>100	>100
Etoposide *^c^*	0.33 ± 0.02	0.58 ± 0.02	1.83 ± 0.09	3.49 ± 0.16	2.23 ± 0.09

*^a^* For cell lines used, see text. *^b^* Results are expressed as mean values ± SD. Each concentration was tested in sextuplicate together with the control and repeated two times in separate experiments. *^c^* Reference compound.

The new compounds as well as the diterpenes used to assemble the dimers were evaluated for antiproliferative effects using five different human cell lines. From the starting diterpenes, compounds **2** and **6 ** showed no antiproliferative effect on any of the cell lines. From the dimeric derivatives containing an ether function, compound **7** showed no antiproliferative effect. The diethers **8–10**, differing in the number of CH_2_ units of the linker, presented better antiproliferative activity with maximum effect for the derivative **9**. The dimer formed by the esterification of compounds **1** and **2** (compound **11**) and its corresponding methyl ester (compound **12**) show the importance of a free carboxylic acid function in the antiproliferative effect. Compound **11** showed activity against MRC-5, AGS and HL-60 cell lines but not against SK-MES-1 and J82 cells. Compound **12** showed effect against SK-MES-1 and J82 cells but was devoid of activity against MRC-5, AGS and HL-60 cell lines. The antiproliferative activity of both diesters with succinic and phthalic acid (compounds **13** and **14**) was similar.

For compounds **15–18**, methylation of the carboxylic acid function at C-19 reduced the antiproliferative effect, as can be seen comparing the pairs **15–16** and **17–18**, respectively. Some selectivity against AGS and HL-60 cells was found for the dimers **15** and **17**, with better effect for compound **17**, differing from **15** in one CH_2_ unit in the triazole linker. On the other hand, the compounds **19** and **20** showed no antiproliferative effect on any of the cell lines, except on HL-60 for compound **19**.

## 3. Experimental

### 3.1. General Procedures

Melting points were determined on a Koffler hot stage apparatus (Electrothermal 9100, Dubuque, IA, USA) and were uncorrected. Optical rotations were measured on a Jasco DIP 370 (Jasco Analytical Instruments, Easton, MD, USA) polarimeter in CHCl_3_ at 20 °C. IR spectra were recorded on a Nicolet Nexus 470 FT-IR instrument (Thermo Electron Corporation, Whaltham, MA, USA). The NMR spectra were recorded on a Bruker Avance 400 (Bruker, Rheinstetten, Germany) spectrometer at 400 MHz for ^1^H and 100 MHz for ^13^C in CDCl_3_. Chemical shifts are given in ppm with TMS as the internal standard. High-resolution mass spectra were measured on a VG Micromass ZAB-2F at 70 eV (Varian Inc., Palo Alto, CA, USA). Merck silica gel (0.063–0.2) was used for column chromatography, pre-coated Si gel plates (Merck, Kieselgel 60 F_254_, 0.25 mm) were used for TLC analysis. TLC spots were visualized by spraying the chromatograms with *p*-anisaldehyde-ethanol-acetic acid-H_2_SO_4_ (2:170:20:10 v/v) and heating at 110 °C for 3 min. Dicyclohexylcarbodiimide (DCC) and dimethylaminopyridine (DMAP) were from Merck (Germany). 4-Toluenesulfonyl chloride (TsCl) from Fluka (St. Louis, MO, USA), propargyl alcohol, 3-butyn-1-ol and 4-pentynoic acid were from Aldrich (Streinheim, Germany). 1,4-dibromobutane, 1,5-dibromopentane and 1,9-dibromononane were from Aldrich (St. Louis, MO, USA). Sodium azide (Sigma-Aldrich, St. Louis, MO, USA), copper (II) sulphatepentahydrate (Aldrich, St. Louis, MO, USA) and sodium ascorbate (Sigma, St. Louis, MO, USA). The diterpene15-hydroxy-labd-8(17)-en-19-oic acid (imbricatolic acid) was isolated from the resin of *Araucaria araucana* as previously described [17,22]. To obtain the starting compound for the synthesis, imbricatolic acid was methylated with diazomethane to afford compound **1**. Compound **2** was obtained from imbricatolic acid by oxidation with Jones reagent (CrO_3_/H_2_SO_4_/H_2_O).

*15-Hydroxyimbricatolic acid methyl ester* (**1**). Imbricatolic acid (3.00 g, 9.32 mmol) was dissolved in a solution of CH_2_N_2_/Et_2_O. The mixture was stirred at room temperature for 3 h, taken to dryness under reduced pressure and purified by flash column chromatography (CC) on silica gel, eluting with hexane/EtOAc (9:1) to yield 1 (2.85 g, 91%): colorless resin; [α]20 *D* +50 (*c* 0.016, CHCl_3_); IR (film) *ν*_max_ 3440, 2937, 2873, 1722, 1426, 1147 cm^−1^; The NMR data are in concordance with those informed previously [22,23]; HREIMS *m/z* 337.2753 [M+H]^+^ (calcd for C_21_H_37_O_3_, 337.2742). 

*Labd-8(17)-en-15,19-dioic acid (junicedric acid)* (**2**). To a solution of imbricatolic acid (3.50 g, 10.54 mmol) in acetone (0.1 M), 3 eq of Jones reagent (1 eq CrO_3_/35 eq H_2_O/1 eq H_2_SO_4_ 16 M) were added at 0 °C. After 10–20 min, the reaction was poured over saturated NaHCO_3_ (50 mL) and extracted with ethyl ether (2 × 50 mL). The ethyl ether solution was washed with brine, dried over anhydrous Na_2_SO_4_, filtered and taken to dryness under reduced pressure. The residue was purified by flash CC on silica gel eluting with hexane/EtOAc (8:2) to yield **2** (2.97 g, 84%): colorless resin; [α]20 *D* +42 (*c* 0.159, CHCl_3_); IR (film) *ν*_max_ 3420, 2937, 2847, 1698, 1644, 1467, 1155 cm^−1^; The NMR data are in concordance with those informed previously [22,23]; HREIMS *m/z* 337.2342 [M+H]^+^ (calcd for C_20_H_33_O_4_, 337.2379).

*15-Azidoimbricatolic acid methyl ester* (**3**). (i) To a solution of compound **1** (2.00 g, 6.21 mmol) in pyridine (0.1 M), a solution of TsCl (1.30 g, 6.83 mmol) was added at 0 °C and the mixture was stirred at room temperature for 24 h. The reaction mixture was cooled in an ice bath, water was added, and the aqueous phase was extracted with EtOAc (3 × 20 mL), dried over anhydrous Na_2_SO_4_ and taken to dryness under reduced pressure. The residue was purified by silica gel CC eluting with hexane/EtOAc (8:2), yielding 2.37 g (78%) of the tosylated compound **1**. (ii) The tosylated compound (2.30 g, 4.70 mmol) and NaN_3_ (0.61 g, 9.40 mmol) in DMF (0.1 M) were stirred at room temperature for 24 h. The reaction mixture was cooled in an ice-bath, water was added, and the aqueous phase was extracted with EtOAc (3 × 20 mL). The organic phase was dried over anhydrous Na_2_SO_4_, taken to dryness and the residue was purified by silica gel CC eluting with hexane/EtOAc (9:1), yielding **3** (1.42 g, 84%): yellow oil; [α]20 *D* +83 (*c* 0.068, CHCl_3_); IR (film) *ν*_max_ 2931, 2843, 2094, 1725, 1644, 1464, 1153 cm^−1^; ^1^H-NMR (CDCl_3_): δ 0.46 (3H, s, H-20), 0.86 (3H, d, *J =* 6.5 Hz, H-16), 0.88 (1H, m, H-12), 1.02 (1H, m, H-3), 1.06 (1H, m, H-1), 1.14 s (3H, s, H-18), 1.21 (1H, m, H-11), 1.25 (1H, m, H-5), 1.34 (1H, m, H-14), 1.42–1.52 (5H, m, H-2, H-9, H-11, H-12 and H-13), 1.60 (1H, m, H-14), 1.72 (1H, m, H-6), 1.77–1.80 (1H, m, H-2), 1.80–1.84 (1H, m, H-1), 1.86–1.89 (1H, m, H-7), 1.90–1.95 (1H, m, H-6), 2.13 (1H, brd, *J =* 14.2 Hz, H-3), 2.36 (1H, dt, *J =* 12.1; 3.0 Hz, H-7), 3.23 (2H, m, H-15), 3.57 (3H, s, OMe), 4.44 (1H, s, H-17), 4.80 (1H, s, H-17); ^13^C-NMR (CDCl_3_): 39.17 (C-1), 19.97 (C-2), 38.26 (C-3), 44.27 (C-4), 56.35 (C-5), 26.25 (C-6), 38.78 (C-7), 148.24 (C-8), 56.54 (C-9), 40.34 (C-10), 21.05 (C-11), 35.93 (C-12), 31.02 (C-13), 35.44 (C-14), 49.51 (C-15), 19.54 (C-16), 106.27 (C-17), 28.79 (C-18), 177.64 (C-19), 12.54 (C-20), 51.05 (OMe); HREIMS *m/z* 362.2796 [M+H]^+^ (calcd for C_21_H_36_N_3_O_2_, 362.2807). 

*15-(4-(Hydroxymethyl)-1H-1,2,3-triazol-1-yl)-imbricatolic acid methyl ester* (**4**). Compound **3** (100 mg, 0.28 mmol) and propargyl alcohol (24 μL, 0.42 mmol), were dissolved in *t*-BuOH/H_2_O (3 mL/3 mL) followed by the addition of CuSO_4_·5H_2_O (7 mg, 0.028 mmol, dissolved in 200 μL of water) and sodium ascorbate (11 mg, 0.056 mmol, dissolved in 200 μL of water). The solution was stirred at room temperature for 24 h. The reaction mixture was cooled in an ice-bath, water was added, and the aqueous phase was extracted with EtOAc (3 × 20 mL). The organic phase was dried over anhydrous Na_2_SO_4_, taken to dryness and the residue was purified by silica gel CC eluting with hexane/EtOAc (8:2) to yield **4** (85 mg, 73%): colorless oil; [α]20 *D* +55 (*c* 0.021, CHCl_3_); IR (film) *ν*_max_ 3420, 2943, 2846, 1725, 1644, 1464, 1153 cm^−1^; ^1^H-NMR (CDCl_3_): δ 0.47 (3H, s, H-20), 0.94 (3H, d, *J =* 6.5 Hz, H-16), 0.95-1.01 (1H, m, H-12), 1.02 (1H, m, H-3), 1.02–1.07 (1H, m, H-1),1.16 s (3H, s, H-18), 1.23 (1H, m, H-11), 1.27 (1H, m, H-5), 1.40 (1H, m, H-14β), 1.48-1.54 (4H, m, H-2, H-9, H-12 and H-13), 1.68 (1H, m, H-14α), 1.73 (1H, m, H-6), 1.77–1.82 (1H, m, H-2), 1.79–1.84 (2H, m, H-1 and H-11), 1.87–1.90 (1H, m, H-7), 1.92–1.98 (1H, m, H-6), 2.15 (1H, brd, *J =* 12.9 Hz, H-3), 2.37 (1H, dt, *J =* 11.9; 3.0 Hz, H-7), 3.59 (3H, s, OMe), 4.32 (2H, m, H-15), 4.43 (1H, s, H-17), 4.76 (2H, s, H-3'), 4.82 (1H, s, H-17), 7.50 (1H, s, H-1'); ^13^C-NMR (CDCl_3_): 39.17 (C-1), 19.95 (C-2), 38.23 (C-3), 44.32 (C-4), 56.34 (C-5), 26.25 (C-6), 38.76 (C-7), 148.25 (C-8), 56.56 (C-9), 40.37 (C-10), 20.98 (C-11), 35.81 (C-12), 31.02 (C-13), 37.11 (C-14), 48.64 (C-15), 19.43 (C-16), 106.30 (C-17), 28.81 (C-18), 177.89 (C-19), 12.56 (C-20), 51.18 (OMe), 121.72 (C-1'), 147.66 (C-2'), 56.05 (C-3'); HREIMS *m/z* 418.3044 [M+H]^+^ (calcd for C_24_H_40_N_3_O_3_, 418.3069). 

*15-(4-(2-Hydroxyethyl)-1H-1,2,3-triazol-1-yl)-imbricatolic acid methyl ester* (**5**). Compound **5** was synthesized as described for **4** starting from compound **3**, using 3-butyn-1-ol instead of propargyl alcohol, to afford 82 mg (68%) of 5: colorless oil; [α]20 *D* +37 (*c* 0.034, CHCl_3_); IR (film) *ν*_max_ 3422, 2946, 2846, 1723, 1644, 1464, 1153 cm^−1^; ^1^H-NMR (CDCl_3_): δ 0.46 (3H, s, H-20), 0.94 (3H, d, *J =* 6.5 Hz, H-16), 0.95–1.01 (1H, m, H-12), 1.02 (1H, m, H-3), 1.02–1.07 (1H, m, H-1), 1.16 (3H, s, H-18), 1.23 (1H, m, H-11), 1.27 (1H, m, H-5), 1.39 (1H, m, H-14β), 1.48–1.53 (4H, m, H-2, H-9, H-12 and H-13), 1.68 (1H, m, H-14α), 1.73 (1H, m, H-6), 1.77–1.82 (1H, m, H-2), 1.79-1.84 (2H, m, H-1 and H-11), 1.87–1.90 (1H, m, H-7), 1.93–1.99 (1H, m, H-6), 2.16 (1H, brd, *J =* 12.9 Hz, H-3), 2.38 (1H, dt, *J =* 11.9; 3.0 Hz, H-7), 2.92 (2H, t, *J =* 5.6 Hz, H-3'), 3.59 (3H, s, OMe), 3.93 (2H, brs, H-4'), 4.32 (2H, m, H-15), 4.42 (1H, s, H-17), 4.81 (1H, s, H-17), 7.37 (1H, s, H-1'); ^13^C-NMR (CDCl_3_): 39.21 (C-1), 19.90 (C-2), 38.27 (C-3), 44.33 (C-4), 56.37 (C-5), 26.27 (C-6), 38.79 (C-7), 148.31 (C-8), 56.57 (C-9), 40.39 (C-10), 21.03 (C-11), 35.83 (C-12), 31.08 (C-13), 37.16 (C-14), 48.50 (C-15), 19.51 (C-16), 106.28 (C-17), 28.85 (C-18), 177.79 (C-19), 12.59 (C-20), 51.17 (OMe), 121.44 (C-1'), 147.60 (C-2'), 28.70 (C-3'), 61.66 (C-4'); HREIMS *m/z* 432.3564 [M+H]^+^ (calcd for C_25_H_42_N_3_O_3_, 432.3226).

*15-(4-(2-Carboxyethyl)-1H-1,2,3-triazol-1-yl)-imbricatolic acid methyl ester* (**6**). **6** was synthesized as described for 4 from 3, using 4-pentynoic acid as the alkyne to afford 91 mg (71%) of **6**: colorless oil; [α]20 *D* +56 (*c* 0.010, CHCl_3_); IR (film) *ν*_max_ 3322, 2940, 2846, 1723, 1644, 1464, 1153 cm^−1^; ^1^H-NMR (CDCl_3_): δ 0.48 (3H, s, H-20), 0.94 (3H, d, *J =* 6.5 Hz, H-16), 0.95–1.01 (1H, m, H-12), 1.03 (1H, m, H-3), 1.02–1.07 (1H, m, H-1), 1.18 (3H, s, H-18), 1.23 (1H, m, H-11), 1.27 (1H, m, H-5), 1.38 (1H, m, H-14β), 1.47–1.55 (4H, m, H-2, H-9, H-12 and H-13), 1.68 (1H, m, H-14α ), 1.73 (1H, m, H-6), 1.77–1.82 (1H, m, H-2), 1.78–1.84 (2H, m, H-1 and H-11), 1.87–1.90 (1H, m, H-7), 1.93–1.99 (1H, m, H-6), 2.16 (1H, brd, *J =* 12.9 Hz, H-3), 2.37 (1H, dt, *J =* 11.9; 3.0 Hz, H-7), 2.79 (2H, t, *J =* 7.0 Hz, H-4'), 3.04 (2H, t, *J =* 7.0 Hz, H-3'), 3.61 (3H, s, OMe), 4.36 (2H, m, H-15), 4.44 (1H, s, H-17), 4.83 (1H, s, H-17), 7.34 (1H, s, H-1'); ^13^C-NMR (CDCl_3_): 39.18 (C-1), 19.96 (C-2), 38.24 (C-3), 44.29 (C-4), 56.32 (C-5), 26.25 (C-6), 38.76 (C-7), 148.30 (C-8), 56.54 (C-9), 40.36 (C-10), 21.01 (C-11), 35.79 (C-12), 31.07 (C-13), 37.13 (C-14), 48.57 (C-15), 19.47 (C-16), 106.26 (C-17), 28.82 (C-18), 177.79 (C-19), 12.56 (C-20), 51.16 (OMe), 121.11 (C-1'), 147.25 (C-2'), 20.65 (C-3'), 33.41 (C-4'), 176.23 (C-5'); HREIMS *m/z* 460.4086 [M+H]^+^ (calcd for C_26_H_42_N_3_O_4_, 460.4048).

*Dimer A* (**7**). To a solution of **1** (150 mg, 0.45 mmol) in DMF (10 mL) was added NaH (16 mg, 0.68 mmol) and tosylated compound **1** (220 mg, 0.45 mmol, for preparation see compound **3**). The mixture was stirred at room temperature for 4 h, cooled in an ice-bath and after addition of water, the product was extracted with EtOAc (3 × 10 mL), washed with brine, and dried over anhydrous Na_2_SO_4_. The residue was purified by flash CC on silica gel eluting with hexane/EtOAc (8:2) to yield **7** (153 mg, 51%): pale yellow oil; [α]20 *D* +41 (*c* 0.009, CHCl_3_); IR (film) *ν*_max_ 2949, 2867, 1723, 1465, 1151 cm^−1^; ^1^H-NMR (CDCl_3_): δ 0.49 (6H, s, 2xH-20), 0.87 (6H, d, *J =* 6.5 Hz, 2 × H-16), 0.90 (2H, m, 2 × H-12), 1.00–1.04 (2H, m, 2 × H-3), 1.03-1.08 (2H, m, 2 × H-1), 1.17 (6H, s, 2 × H-18), 1.23–1.28 (2H, m, 2 × H-11), 1.26–1.33 (2H, m, 2 × H-5), 1.37 (2H, m, 2 × H-14β), 1.46–1.54 (10H, m, 2 × H-2, 2 × H-9, 2 × H-11, 2 × xH-12 and 2 × H-13), 1.63 (2H, m, 2 × H-14α), 1.75–1.80 (4H, m, 2 × H-2 and 2 × H-6), 1.80–1.85 (2H, m, 2 × H-1), 1.84–1.90 (2H, m, 2 × H-7), 1.91–1.97 (2H, m, 2 × H-6), 2.13 (2H, brd, *J =* 14.2 Hz, 2 × H-3), 2.38 (2H, dt, *J =* 12.1; 3.0 Hz, 2 × H-7), 3.40 (4H, t, *J =* 7.0 Hz, 2 × H-15), 3.61 (6H, s, 2 × OMe), 4.48 (2H, s, 2 × H-17), 4.82 (2H, s, 2 × H-17); ^13^C-NMR (CDCl_3_): see Table 1; HREIMS *m/z* 655.5412 [M+H]^+^ (calcd for C_42_H_71_O_5_, 655.5301). 

*Dimer B* (**8**). To a solution of **1** (150 mg, 0.45 mmol) in DMF (10 mL) was added NaH (16 mg, 0.68 mmol) and 1,4-dibromobutane (54 *μ*L, 0.45 mmol). The mixture was stirred at room temperature for 4 h, cooled in an ice-bath and after addition of water, the product was extracted with EtOAc (3 × 10 mL), washed with brine, and dried over anhydrous Na_2_SO_4_. The residue was purified by flash CC on silica gel eluting with hexane/EtOAc (9:1) to yield **7** (78 mg, 48%): pale yellow oil; [α]20 *D* +32 (*c* 0.037; CHCl_3_); IR (film) *ν*_max_ 2942, 2864, 1723, 1449, 1151 cm^−1^; ^1^H-NMR (CDCl_3_): δ 0.49 (6H, s, 2 × H-20), 0.87 (6H, d, *J =* 6.5 Hz, 2 × H-16), 0.88 (2H, m, 2 × H-12), 1.00-1.04 (2H, m, 2 × H-3), 1.03–1.08 (2H, m, 2 × H-1), 1.17 (6H, s, 2 × H-18), 1.24–1.30 (2H, m, 2 × H-11), 1.27–1.34 (2H, m, 2 × H-5),1.34 (2H, m, 2 × H-14β), 1.45–1.53 (10H, m, 2 × H-2, 2 × H-9, 2 × H-11, 2 × H-12 and 2 × H-13), 1.63 (2H, m, 2 × H-14α), 1.75–1.80 (4H, m, 2 × H-2 and 2 × H-6), 1.80–1.85 (2H, m, 2 × H-1), 1.84–1.89 (2H, m, 2 × H-7), 1.88–1.95 (2H, m, 2 × H-6 and 4H, m, 2 × H-2''), 2.15 (2H, brd, *J =* 14.2 Hz, 2 × H-3), 2.38 (2H, dt, *J =* 12.1; 3.0 Hz, 2 × H-7), 3.40 (4H, m, 2 × H-1''), 3.43 (4H, t, *J =* 7.0 Hz, 2 × H-15), 3.60 (6H, s, 2 × OMe), 4.48 (2H, s, 2 × H-17), 4.82 (2H, s, 2 × H-17); ^13^C-NMR (CDCl_3_): see Table 1; HREIMS *m/z* 727.5793 [M+H]^+^ (calcd for C_46_H_79_O_6_, 727.5876). 

*Dimer C* (**9**). **9** was synthesized as described for **8** from **1** using 1,5-dibromopentane to afford 93 mg (56%) of **9**: colorless oil; [α]20 *D* +35 (*c* 0.030, CHCl_3_); IR (film) *ν*_max_ 2942, 2864, 1723, 1468, 1148 cm^−1^; ^1^H-NMR (CDCl_3_): δ 0.49 (6H, s, 2 × H-20), 0.87 (6H, d, *J =* 6.5 Hz, 2 × H-16), 0.90 (2H, m, 2 × H-12), 1.00–1.04 (2H, m, 2 × H-3), 1.02–1.06 (2H, m, 2 × H-1), 1.17 (6H, s, 2 × H-18), 1.24–1.31 (2H, m, 2 × H-11), 1.27–1.33 (2H, m, 2 × H-5), 1.34 (2H, m, 2 × H-14β), 1.45–1.55 (10H, m, 2 × H-2, 2 × H-9, 2 × H-11, 2 × H-12, 2 × H-13 and 2H, m, H-3''), 1.62 (2H, m, 2 × H-14α), 1.75-1.80 (4H, m, 2 × H-2 and 2 × H-6), 1.79–1.84 (2H, m, 2 × H-1), 1.84–1.89 (2H, m, 2 × H-7 and 4H, m, 2 × H-2''), 1.91–1.96 (2H, m, 2 × H-6), 2.16 (2H, brd, *J =* 14.2 Hz, 2 × H-3), 2.39 (2H, dt, *J =* 12.1; 3.0 Hz, 2 × H-7), 3.39 (4H, m, 2 × H-1''), 3.40 (4H, t, *J =* 7.0 Hz, 2 × H-15), 3.60 (6H, s, 2xOMe), 4.48 (2H, s, 2 × H-17), 4.82 (2H, s, 2 × H-17); ^13^C-NMR (CDCl_3_): see Table 1; HREIMS *m/z* 741.6012 [M+H]^+^ (calcd for C_47_H_81_O_6_, 741.6033). 

*Dimer D* (**10**). **10** was synthesized as described for **8** from **1** using 1,9-dibromononane to afford 73 mg (41%) of **10**: yellow oil; [α]20 *D* +20 (*c* 0.098, CHCl_3_); IR (film) *ν*_max_ 2923, 2849, 1723, 1462, 1152 cm^−1^; ^1^H-NMR (CDCl_3_): δ 0.47 (6H, s, 2 × H-20), 0.86 (6H, d, *J =* 6.5 Hz, 2 × H-16), 0.89 (2H, m, 2 × H-12), 1.00–1.04 (2H, m, 2 × H-3), 1.01–1.05 (2H, m, 2 × H-1), 1.16 (6H, s, 2 × H-18), 1.24–1.30 (2H, m, 2 × H-11 and 6H, m, 2 × H-4'' and H-5''), 1.27–1.34 (2H, m, 2 × H-5), 1.34 (2H, m, 2 × H-14β), 1.47–1.55 (10H, m, 2 × H-2, 2 × H-9, 2 × H-11, 2 × H-12, 2 × H-13 and 4H, m, 2 × H-3''), 1.62 (2H, m, 2 × H-14α), 1.75–1.80 (4H, m, 2 × H-2 and 2 × H-6), 1.81–1.85 (2H, m, 2 × H-1 and 4H, m, 2 × H-2''), 1.86–1.90 (2H, m, 2 × H-7), 1.92–1.97 (2H, m, 2 × H-6), 2.14 (2H, brd, *J =* 14.2 Hz, 2 × H-3), 2.38 (2H, dt, *J =* 12.1; 3.0 Hz, 2 × H-7), 3.36 (4H, m, 2 × H-1''), 3.38 (4H, t, *J =* 7.0 Hz, 2 × H-15), 3.60 (6H, s, 2xOMe), 4.47 (2H, s, 2 × H-17), 4.80 (2H, s, 2 × H-17); ^13^C-NMR (CDCl_3_): see Table 1; HREIMS *m/z* 797.6721 [M+H]^+^ (calcd for C_51_H_89_O_6_, 797.6659). 

*Dimer E* (**11**). **2** (120 mg, 0.36 mmol), DCC (111 mg, 0.54 mmol), catalytic amount of DMAP and **1** (120 mg, 0.36 mmol) in dry CH_2_Cl_2_ (10 mL) were stirred at room temperature for 2 h. The reaction mixture was cooled in an ice-bath, water was added, and the aqueous phase was extracted with EtOAc (3 × 20 mL), and dried over anhydrous Na_2_SO_4_, taken to dryness and the residue was purified by silica gel CC eluting with hexane/EtOAc (8:2), yielding **11** (153 mg, 65%): colorless oil; [α]20 *D* +34 (*c* 0.148, CHCl_3_); IR (film) *ν*_max_ 3322, 2930, 2847, 1724, 1644, 1465, 1152 cm^−1^; ^1^H-NMR (CDCl_3_): δ 0.46 (3H, s, H-20), 0.55 (3H, s, H-20'), 0.86 (3H, d, *J =* 6.5 Hz, H-16), 0.89 (3H, d, *J =* 6.5 Hz, H-16'), 0.86-0.91 (2H, m, H-12 and H-12'), 0.98–1.08 (4H, m, H-1, H-1', H-3 and H-3'), 1.14 (3H, s, H-18), 1.19 (3H, s, H-18'), 1.20–1.25 (4H, m, H-5, H-5', H-11 and H-11'), 1.34 (1H, m, H-14β), 1.45–1.50 (10H, m, H-2, H-2', H-9, H-9', H-11, H-11', H-12, H-12', H-13 and H-13'), 1.64 (1H, m, H-14α), 1.70–1.75 (2H, m, H-6 and H-6'), 1.76-1.84 (4H, m, H-1, H-1', H-2 and H-2'), 1.86–1.93 (4H, m, H-6, H-6', H-7 and H-7'), 2.04 (1H, dd, *J =* 14.5; 8.2 Hz, H-14'β), 2.13 (2H, m, H-3 and H-3'), 2.27 (1H, dd, *J =* 14.5; 5.9 Hz, H-14'α), 2.36 (2H, m, H-7 and H-7'), 3.57 (3H, s, OMe), 4.04 (4H, m, H-15 and H-15'), 4.44 (2H, s, H-17 and H-17'), 4.79 (2H, s, H-17 and H-17'); ^13^C-NMR (CDCl_3_): see Table 2; HREIMS *m/z* 655.4924 [M+H]^+^ (calcd for C_41_H_67_O_6_, 655.4937). 

*Dimer F* (**12**). Compound **11** (100 mg, 0.15 mmol), was methylated with a solution of CH_2_N_2_ in ethyl ether, yielding 92 mg (92%) of **12**: colorless oil; [α]20 *D* +45 (*c* 0.029, CHCl_3_); IR (film) *ν*_max_ 2949, 2842, 1723, 1644, 1465, 1152 cm^−1^; ^1^H-NMR (CDCl_3_): δ 0.48 (6H, s, H-20 and H-20'), 0.89 (3H, d, *J =* 6.5 Hz, H-16), 0.92 (3H, d, *J =* 6.5 Hz, H-16'), 0.88–0.93 (2H, m, H-12 and H-12'), 1.00–1.08 (4H, m, H-1, H-1', H-3 and H-3'), 1.17 (6H, s, H-18 and H-18'), 1.22–1.30 (4H, m, H-5, H-5', H-11 and H-11'), 1.36 (1H, m, H-14β), 1.46–1.52 (10H, m, H-2, H-2', H-9, H-9', H-11, H-11', H-12, H-12', H-13 and H-13'), 1.65 (1H, m, H-14α), 1.72–1.78 (2H, m, H-6 and H-6'), 1.80–1.87 (4H, m, H-1, H-1', H-2 and H-2'), 1.88–1.95 (4H, m, H-6, H-6', H-7 and H-7'), 2.06 (1H, dd, *J =* 14.5; 8.3 Hz, H-14'β), 2.14 (2H, m, H-3 and H-3'), 2.29 (1H, dd, *J =* 14.5; 5.9 Hz, H-14'α), 2.38 (2H, m, H-7 and H-7'), 3.60 (6H, s, OMe and OMe'), 4.06 (4H, m, H-15 and H-15'), 4.46 (2H, s, H-17 and H-17'), 4.82 (2H, s, H-17 and H-17'); ^13^C-NMR (CDCl_3_): see Table 2; HREIMS *m/z* 669.5131 [M+H]^+^ (calcd for C_42_H_69_O_6_, 669.5094). 

*Dimer G* (**13**). **13** was synthesized as described for **11** from **1** using succinic acid to afford 76 mg (45%) of **13**: pale yellow oil; [α]20 *D* +34 (*c* 0.042, CHCl_3_); IR (film) *ν*_max_ 3320, 2949, 2841, 1721, 1645, 1465, 1155 cm^−1^; ^1^H-NMR (CDCl_3_): δ 0.49 (6H, s, 2 × H-20), 0.89 (6H, d, *J =* 6.5 Hz, 2 × H-16), 0.87–0.93 (2H, m, 2x H-12), 0.99–1.07 (4H, m, 2 × H-1 and 2 × H-3), 1.18 (6H, s, 2 × H-18), 1.23–1.29 (4H, m, 2 × H-5 and 2 × H-11), 1.30 (2H, m, 2 × H-14β), 1.46–1.52 (10H, m, 2 × H-2, 2 × H-9, 2 × H-11, 2 × H-12 and 2 × H-13), 1.66 (2H, m, 2 × H-14α), 1.70–1.76 (2H, m, 2 × H-6), 1.77–1.84 (4H, m, 2 × H-1 and 2 × H-2), 1.86–1.94 (4H, m, 2 × H-6 and 2 × H-7), 2.16 (2H, m, 2 × H-3), 2.38 (2H, m, 2 × H-7), 2.64 (4H, brd, *J =* 6.1 Hz, 2 × H-2''), 3.61 (6H, s, 2 × OMe), 4.11 (4H, m, 2 × H-15), 4.47 (2H, s, 2 × H-17), 4.82 (2H, s, 2 × H-17); ^13^C-NMR (CDCl_3_): see Table 1; HREIMS *m/z* 755.5546 [M+H]^+^ (calcd for C_46_H_75_O_8_, 755.5462). 

*Dimer H* (**14**). **14** was synthesized as described for **11** from **1** using phthalic acid to afford 95 mg (53%) of 14: yellow oil; [α]20 *D* +33 (*c* 0.053, CHCl_3_); IR (film) *ν*_max_ 2949, 2842, 1723, 1644, 1465, 1148 cm^−1^; ^1^H-NMR (CDCl_3_): δ 0.42 (6H, s, 2 × H-20), 0.88 (6H, d, *J =* 6.5 Hz, 2 × H-16), 0.88–0.95 (2H, m, 2 × H-12), 0.97–1.06 (4H, m, 2 × H-1 and 2 × H-3), 1.11 (6H, s, 2 × H-18), 1.20–1.25 (4H, m, 2 × H-5 and 2 × H-11), 1.30 (2H, m, 2 × H-14β), 1.40–1.48 (10H, m, 2 × H-2, 2 × H-9, 2 × H-11, 2 × H-12 and 2 × H-13), 1.68 (2H, m, 2 × H-14α), 1.70–1.76 (2H, m, 2 × H-6), 1.76–1.82 (4H, m, 2 × H-1 and 2 × H-2), 1.84–1.92 (4H, m, 2 × H-6 and 2 × H-7), 2.08 (2H, m, 2 × H-3), 2.33 (2H, m, 2 × H-7), 3.55 (6H, s, 2 × OMe), 4.28 (4H, m, 2 × H-15), 4.42 (2H, s, 2 × H-17), 4.76 (2H, s, 2 × H-17), 7.58 (2H, m, 2 × H-4''), 7.76 (2H, m, 2 × H-3''); ^13^C-NMR (CDCl_3_): see Table 1; HREIMS *m/z* 803.5437 [M+H]^+^ (calcd for C_50_H_74_O_8_, 803.5462). 

*Dimer I* (**15**). (i) **2** (120 mg, 0.36 mmol), DCC (111 mg, 0.54 mmol), catalytic amount of DMAP and propargyl alcohol (31 μL, 0.54 mmol) in dry CH_2_Cl_2_ (10 mL) were stirred at room temperature for 2 h. Yielding the propargyl ester of **2** (92 mg, 66%) after the extraction and purification. (ii) **3** (76 mg, 0.21 mmol) and propargyl ester of **2** (80 mg, 0. 21 mmol), were dissolved in *t*-BuOH/H_2_O (3 mL/3 mL) followed by the addition of 5 mg CuSO_4_.5H_2_O (0.021 mmol, dissolved in 200 μL of water) and 8 mg of sodium ascorbate (0.042 mmol, dissolved in 200 μL of water). The solution was stirred at room temperature for 24 h. The reaction mixture was cooled in an ice-bath, water was added, and the aqueous phase was extracted with EtOAc (3 × 20 mL), and dried over anhydrous Na_2_SO_4_, taken to dryness and the residue was purified by silica gel CC eluting with hexane/EtOAc (8:2), yielding **15** (120 mg, 78%): yellow oil; [α]20 *D* +37 (*c* 0.075, CHCl_3_); IR (film) *ν*_max_ 3320, 2936, 2842, 1718, 1641, 1466, 1152 cm^−1^; ^1^H-NMR (CDCl_3_): δ 0.44 (3H, s, H-20), 0.54 (3H, s, H-20'), 0.85 (3H, d, *J =* 6.5 Hz, H-16), 0.90 (3H, d, *J =* 6.5 Hz, H-16'), 0.94–0.99 (2H, m, H-12 and H-12'), 0.98–1.07 (4H, m, H-1, H-1', H-3 and H-3'), 1.13 (3H, s, H-18), 1.18 (3H, s, H-18'), 1.20–1.28 (4H, m, H-5, H-5', H-11 and H-11'), 1.34 (1H, m, H-14β), 1.45–1.51 (10H, m, H-2, H-2', H-9, H-9', H-11, H-11', H-12, H-12', H-13 and H-13'), 1.65 (1H, m, H-14α), 1.72–1.78 (2H, m, H-6 and H-6'), 1.79–1.85 (4H, m, H-1, H-1', H-2 and H-2'), 1.86–1.96 (4H, m, H-6, H-6', H-7 and H-7'), 2.05 (1H, dd, *J =* 14.8; 8.5 Hz, H-14'β), 2.11 (2H, m, H-3 and H-3'), 2.29 (1H, dd, *J =* 14.8; 5.6 Hz, H-14'α), 2.33 (2H, m, H-7 and H-7'), 3.56 (3H, s, OMe), 4.30 (2H, m, H-15), 4.39 (2H, s, H-17 and H-17'), 4.75 (1H, s, H-17'), 4.78 (1H, s, H-17), 5.16 (2H, s, H-3''), 7.55 (1H, s, H-1''); ^13^C-NMR (CDCl_3_): see Table 2; HREIMS *m/z* 736.5359 [M+H]^+^ (calcd for C_44_H_70_N_3_O_6_, 736.5265). 

*Dimer J* (**16**). Compound **15** (60 mg, 0.08 mmol), was methylated with a solution of CH_2_N_2_ in ethyl ether, yielding 57 mg (94%) of **16**: colorless oil; [α]20 *D* +40 (*c* 0.008, CHCl_3_); IR (film) *ν*_max_ 2929, 2835, 1720, 1643, 1454, 1153 cm^−1^; ^1^H-NMR (CDCl_3_): δ 0.46 (6H, s, H-20 and H-20'), 0.84 (3H, d, *J =* 6.5 Hz, H-16), 0.88 (3H, d, *J =* 6.5 Hz, H-16'), 0.93–0.98 (2H, m, H-12 and H-12'), 0.99–1.06 (4H, m, H-1, H-1', H-3 and H-3'), 1.12 (3H, s, H-18), 1.19 (3H, s, H-18'), 1.20–1.28 (4H, m, H-5, H-5', H-11 and H-11'), 1.33 (1H, m, H-14β), 1.49–1.53 (10H, m, H-2, H-2', H-9, H-9', H-11, H-11', H-12, H-12', H-13 and H-13'), 1.63 (1H, m, H-14α), 1.70–1.77 (2H, m, H-6 and H-6'), 1.79–1.86 (4H, m, H-1, H-1', H-2 and H-2'), 1.88–1.98 (4H, m, H-6, H-6', H-7 and H-7'), 2.02 (1H, dd, *J =* 14.8; 8.5 Hz, H-14'β), 2.12 (2H, m, H-3 and H-3'), 2.28 (1H, dd, *J =* 14.8; 5.6 Hz, H-14'α), 2.34 (2H, m, H-7 and H-7'), 3.59 (6H, s, OMe and OMe'), 4.33 (2H, m, H-15), 4.42 (2H, s, H-17 and H-17'), 4.79 (1H, s, H-17'), 4.81 (1H, s, H-17), 5.18 (2H, s, H-3''), 7.55 (1H, s, H-1''); ^13^C-NMR (CDCl_3_): see Table 2; HREIMS *m/z* 750.5205 [M+H]^+^ (calcd for C_45_H_72_N_3_O_6_, 750.5421). 

*Dimer K* (**17**). **17** was synthesized as described for **15** (i) from **2** using 3-butyn-1-ol to afford 84 mg (58%) of the butyn ester of **2**. (ii) from **3** using butyn ester of **2** to afford 115 mg (74%) of **17**: colorless oil; [α]20 *D* +27 (*c* 0.122, CHCl_3_); IR (film) *ν*_max_ 3315, 2933, 2864, 1723, 1638, 1462, 1152 cm^−1^; ^1^H-NMR (CDCl_3_): δ 0.44 (3H, s, H-20), 0.55 (3H, s, H-20'), 0.85 (3H, d, *J =* 6.5 Hz, H-16), 0.90 (3H, d, *J =* 6.5 Hz, H-16'), 0.92–0.98 (2H, m, H-12 and H-12'), 0.99–1.07 (4H, m, H-1, H-1', H-3 and H-3'), 1.14 (3H, s, H-18), 1.19 (3H, s, H-18'), 1.20–1.28 (4H, m, H-5, H-5', H-11 and H-11'), 1.34 (1H, m, H-14β), 1.45–1.51 (10H, m, H-2, H-2', H-9, H-9', H-11, H-11', H-12, H-12', H-13 and H-13'), 1.64 (1H, m, H-14α), 1.71–1.77 (2H, m, H-6 and H-6'), 1.78–1.84 (4H, m, H-1, H-1', H-2 and H-2'), 1.86–1.95 (4H, m, H-6, H-6', H-7 and H-7'), 2.03 (1H, dd, *J =* 14.8; 8.5 Hz, H-14'β), 2.11 (2H, m, H-3 and H-3'), 2.28 (1H, dd, *J =* 14.8; 5.6 Hz, H-14'α), 2.33 (2H, m, H-7 and H-7'), 3.02 (2H, t, *J =* 6.5 Hz, H-3''), 3.57 (3H, s, OMe), 4.29 (2H, t, *J =* 6.5 Hz, H-4''), 4.31 (2H, m, H-15), 4.40 (1H, s, H-17'), 4.42 (1H, s, H-17), 4.79 (2H, s, H-17 and H-17'), 7.32 (1H, s, H-1''); ^13^C-NMR (CDCl_3_): see Table 2; HREIMS *m/z* 750.5205 [M+H]^+^ (calcd for C_45_H_72_N_3_O_6_, 750.5421). 

*Dimer L* (**18**). Compound **17** (60 mg, 0.08 mmol), was methylated with a solution of CH_2_N_2_ in ethyl ether, yielding 54 mg (88%) of **18**: colorless oil; [α]20 *D* +41 (*c* 0.024, CHCl_3_); IR (film) *ν*_max_ 2947, 2839, 1720, 1643, 1463, 1149 cm^−1^; ^1^H-NMR (CDCl_3_): δ 0.47 (6H, s, H-20 and H-20'), 0.89 (3H, d, *J =* 6.5 Hz, H-16), 0.92 (3H, d, *J =* 6.5 Hz, H-16'), 0.94–1.00 (2H, m, H-12 and H-12'), 1.01–1.09 (4H, m, H-1, H-1', H-3 and H-3'), 1.17 (6H, s, H-18 and H-18'), 1.22–1.30 (4H, m, H-5, H-5', H-11 and H-11'), 1.35 (1H, m, H-14β), 1.46–1.53 (10H, m, H-2, H-2', H-9, H-9', H-11, H-11', H-12, H-12', H-13 and H-13'), 1.65 (1H, m, H-14α), 1.71–1.77 (2H, m, H-6 and H-6'), 1.79–1.86 (4H, m, H-1, H-1', H-2 and H-2'), 1.87–1.93 (4H, m, H-6, H-6', H-7 and H-7'), 2.06 (1H, dd, *J =* 14.8; 8.5 Hz, H-14'β), 2.12 (2H, m, H-3 and H-3'), 2.30 (1H, dd, *J =* 14.8; 5.6 Hz, H-14'α), 2.35 (2H, m, H-7 and H-7'), 3.05 (2H, t, *J =* 6.5 Hz, H-3''), 3.60 (6H, s, OMe and OMe'), 4.32 (2H, t, *J =* 6.5 Hz, H-4''), 4.33 (2H, m, H-15), 4.43 (1H, s, H-17'), 4.44 (1H, s, H-17), 4.82 (2H, s, H-17 and H-17'), 7.32 (1H, s, H-1''); ^13^C-NMR (CDCl_3_): see Table 2; HREIMS *m/z* 764.5391 [M+H]^+^ (calcd for C_46_H_74_N_3_O_6_, 764.5577). 

*Dimer M* (**19**). **19** was synthesized as described for 15 (i) from imbricatolic acid using 5-pentynoic acidto afford 97 mg (62%) of the pentynoic ester of imbricatolic acid. (ii) from **3** using pentynoic ester of imbricatolic acid to afford 107 mg (57%) of **19**: yellow oil; [α]20 *D* +28 (*c* 0.046, CHCl_3_); IR (film) *ν*_max_ 3318, 2947, 2840, 1720, 1639, 1463, 1149 cm^−1^; ^1^H-NMR (CDCl_3_): δ 0.48 (3H, s, H-20), 0.59 (3H, s, H-20'), 0.88 (3H, d, *J =* 6.5 Hz, H-16), 0.93 (3H, d, *J =* 6.5 Hz, H-16'), 0.95–0.99 (2H, m, H-12 and H-12'), 1.00–1.07 (4H, m, H-1, H-1', H-3 and H-3'), 1.17 (3H, s, H-18), 1.23 (3H, s, H-18'), 1.21–1.29 (4H, m, H-5, H-5', H-11 and H-11'), 1.32–1.36 (2H, m, H-14β and H-14'β), 1.47–1.52 (10H, m, H-2, H-2', H-9, H-9', H-11, H-11', H-12, H-12', H-13 and H-13'), 1.65 (2H, m, H-14α and H-14'α), 1.74–1.79 (2H, m, H-6 and H-6'), 1.80–1.86 (4H, m, H-1, H-1', H-2 and H-2'), 1.87–1.95 (4H, m, H-6, H-6', H-7 and H-7'), 2.15 (2H, m, H-3 and H-3'), 2.39 (2H, m, H-7 and H-7'), 2.70 (2H, t, *J =* 7.3 Hz, H-4''), 3.02 (2H, t, *J =* 7.3 Hz, H-3''), 3.60 (3H, s, OMe), 4.08 (2H, m, H-15'), 4.30 (2H, m, H-15),4.43 (1H, s, H-17'), 4.46 (1H, s, H-17), 4.81 (1H, s, H-17'), 4.82 (1H, s, H-17), 7.31 (1H, s, H-1''); ^13^C-NMR (CDCl_3_): see Table 2; HREIMS *m/z* 764.5516 [M+H]^+^ (calcd for C_46_H_74_N_3_O_6_, 764.5577).

*Dimer N* (**20**). Compound **19** (50 mg, 0.065 mmol), was methylated with a solution of CH_2_N_2_ in ethyl ether, yielding 43 mg (86%) of **20**: pale yellow oil; [α]20 *D* +30 (*c* 0.047, CHCl_3_); IR (film) *ν*_max_ 2947, 2860, 1724, 1639, 1463, 1149 cm^−1^; ^1^H-NMR (CDCl_3_): δ 0.43 (3H, s, H-20), 0.44 (3H, s, H-20'), 0.84 (3H, d, *J =* 6.5 Hz, H-16), 0.89 (3H, d, *J =* 6.5 Hz, H-16'), 0.92–0.97 (2H, m, H-12 and H-12'), 0.98–1.05 (4H, m, H-1, H-1', H-3 and H-3'), 1.13 (6H, s, H-18 and H-18'), 1.20–1.27 (4H, m, H-5, H-5', H-11 and H-11'), 1.30–1.35 (2H, m, H-14β and H-14'β), 1.45–1.51 (10H, m, H-2, H-2', H-9, H-9', H-11, H-11', H-12, H-12', H-13 and H-13'), 1.66 (2H, m, H-14α and H-14'α), 1.73–1.78 (2H, m, H-6 and H-6'), 1.79–1.84 (4H, m, H-1, H-1', H-2 and H-2'), 1.86–1.93 (4H, m, H-6, H-6', H-7 and H-7'), 2.11 (2H, m, H-3 and H-3'), 2.34 (2H, m, H-7 and H-7'), 2.66 (2H, t, *J =* 7.3 Hz, H-4''), 2.97 (2H, t, *J =* 7.3 Hz, H-3''), 3.56 (6H, s, OMe and OMe'), 4.04 (2H, m, H-15'), 4.27 (2H, m, H-15), 4.39 (1H, s, H-17'), 4.41 (1H, s, H-17), 4.78 (2H, s, H-17 and H-17'), 7.30 (1H, s, H-1''); ^13^C-NMR (CDCl_3_): see Table 2; HREIMS *m/z* 778.5566 [M+H]^+^ (calcd for C_46_H_73_N_3_O_6_, 778.5734).

### 3.2. Antiproliferative Assay

All human cell lines used in this work were purchased from the American Type Culture Collection (ATCC, Manasas, VA, USA). Normal lung MRC-5 fibroblasts (CCL-171), SK-MES-1 lung cancer cells (HTB-58) and J82 bladder carcinoma cells (HTB-1) were grown as monolayers in minimum essential Eagle medium (MEM) with Earles’s salts, 2 *m*M L-glutamine and 1.5 g/L sodium bicarbonate. Gastric adenocarcinoma AGS cells (CRL-1739) were grown as monolayers in Ham F-12 medium containing 1 *m*M L-glutamine and 1.5 g/L sodium bicarbonate. Promyelocytic leukemia HL-60 cells (CCL-240) were grown in suspension in RPM1 medium containing 1 *m*M sodium pyruvate and 2.0 g/L sodium bicarbonate. All media were supplemented with 10% heat-inactivated FBS, 100 IU/mL penicillin and 100 *µ*g/mL streptomycin. Cells were grown in a humidified incubator with 5% CO_2_ in air at 37 °C. For the antiproliferative assay, adherent cells were plated at a density of 5 × 10^4^ cells/mL and HL-60 cells at 30 × 10^4^ cells/mL. Cells were seeded in 96-well plates (100 *µ*L/well). One day after seeding, cells were treated with medium containing the compounds at concentrations ranging from 0 up to 100 *µ*M during 3 days. The compounds were dissolved in DMSO (1% final concentration) and complete medium. Untreated cells (medium containing 1% DMSO) were used as 100% viability controls. Etoposide (98% purity, Sigma-Aldrich, St. Louis, MO, USA) was used as reference compound. Each concentration was tested in sextuplicate and experiments were repeated 2 times. Cell viability was determined by means of the MTT reduction assay at the end of the incubation with the products. The results were transformed to percentage of controls and the IC_50_ values were graphically obtained from the dose-response curves.

## 4. Conclusions

In the present work, some 14 new dimeric labdane diterpenes were synthesized starting from the natural product imbricatolic acid. Dimeric labdanes are uncommon in Nature and this report presents a suitable methodology for the synthesis of new dimeric terpenes to be assessed for selected bioactivities. The new compounds showed low to moderate antiproliferative effects on the selected cell lines, with some selectivity. Additional studies using other biological models are needed to disclose the potential of the novel dimers as bioactive agents.

## Supplementary Materials

Supplementary materials can be accessed at: http://www.mdpi.com/1420-3049/18/5/5936/s1.
