# Supplementary material for: Dimeric Labdane Diterpenes: Synthesis and Antiproliferative Effects
Source: Molecules. 2013 May 21;18(5):5936–53. doi: 10.3390/molecules18055936 (PMC6270601; doi:10.3390/molecules18055936)

Supplementary Materials

Figure S1. ^{1}H-NMR of Compound **3**.

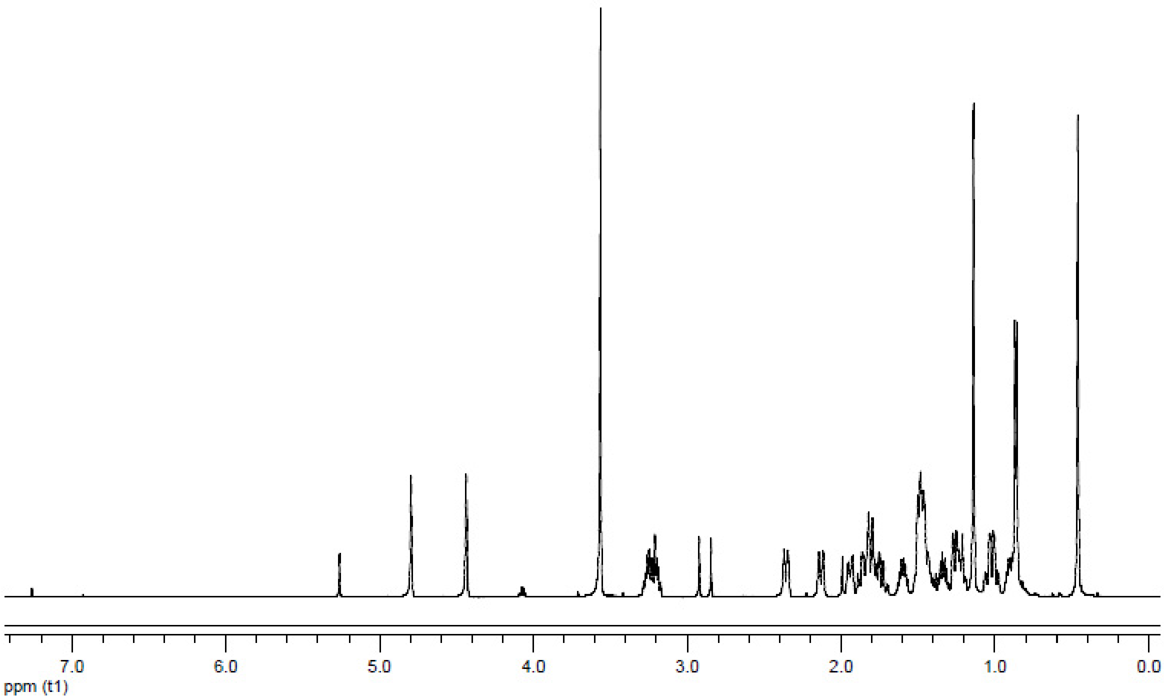

Figure S2. ^{13}C-NMR of Compound **3**.

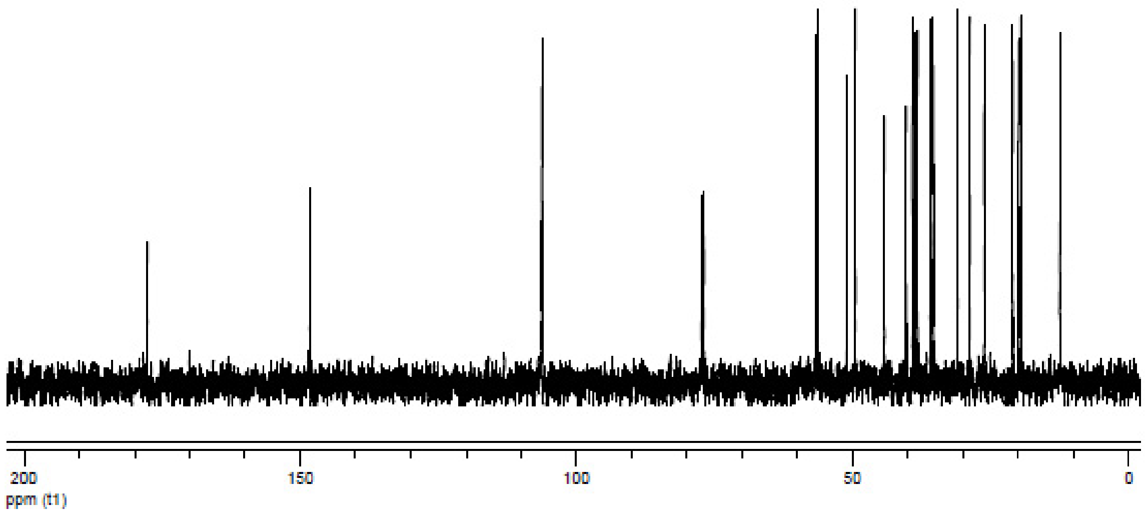

Figure S3. HMQC of Compound **3**.

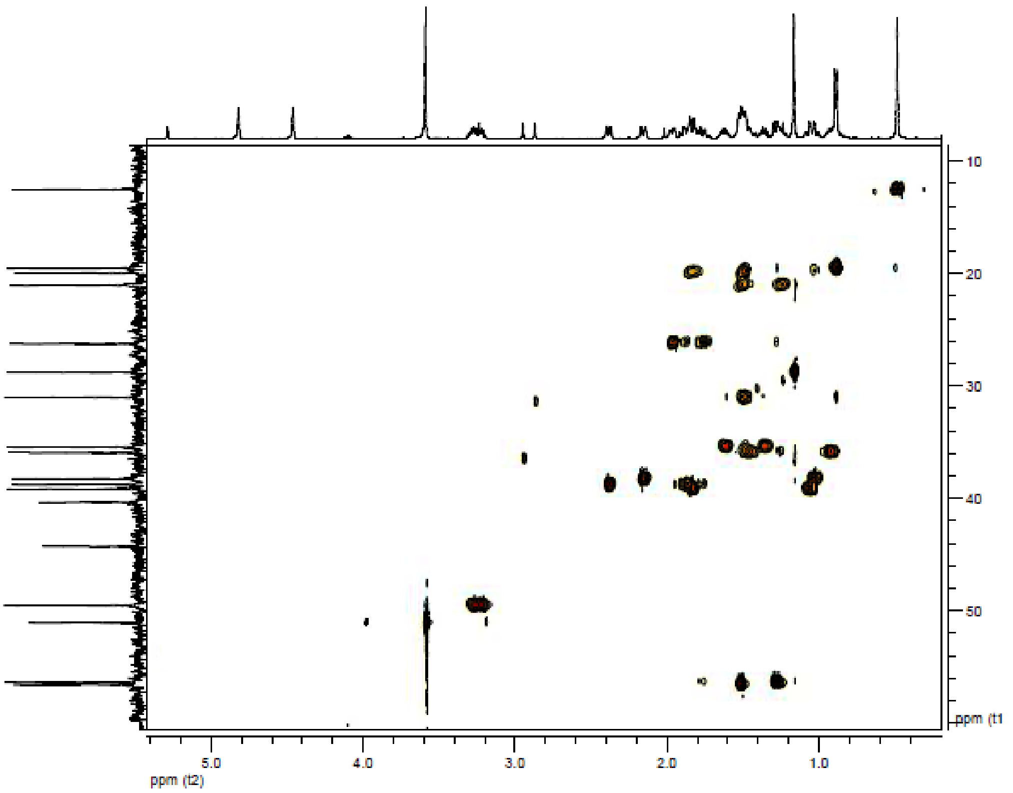

Figure S4. HMBC of Compound **3**.

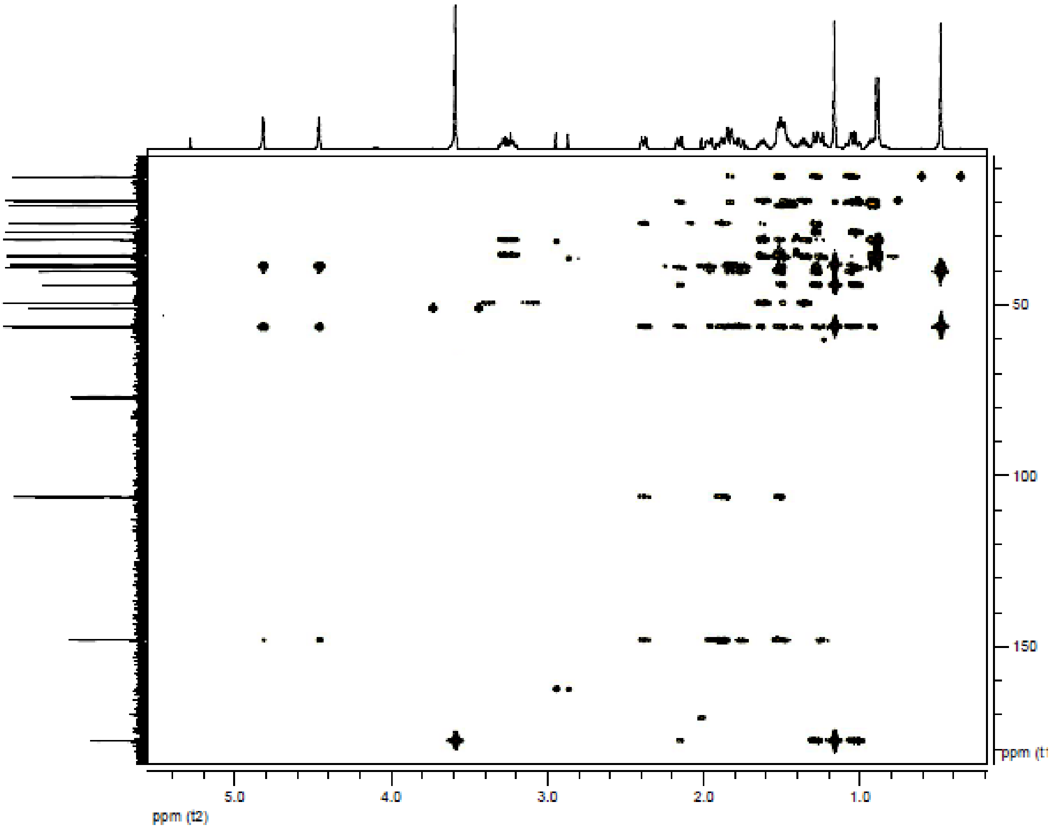

Figure S5. ^{1}H-NMR of Compound **7**.

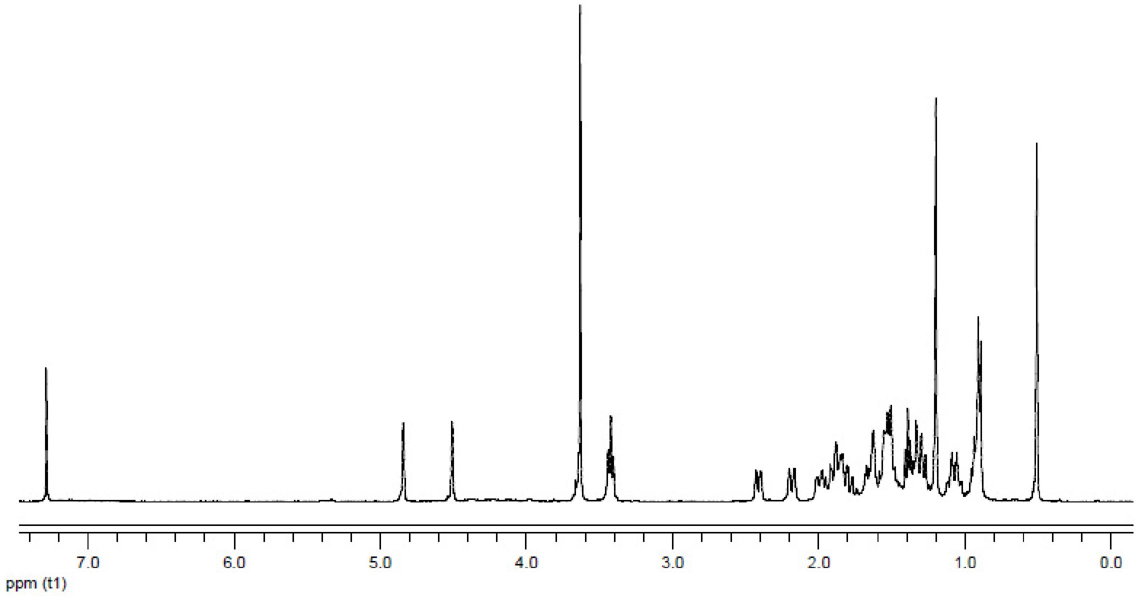

Figure S6. ^{1}H-NMR of Compound **9**.

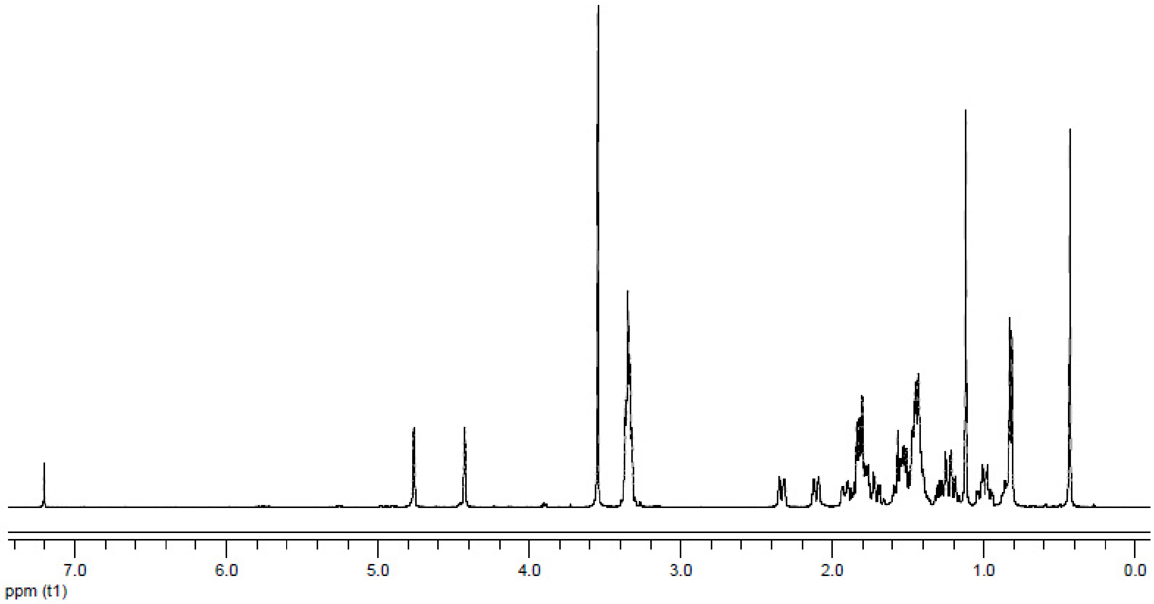

Figure S7. ^{13}C-NMR of Compound **9**.

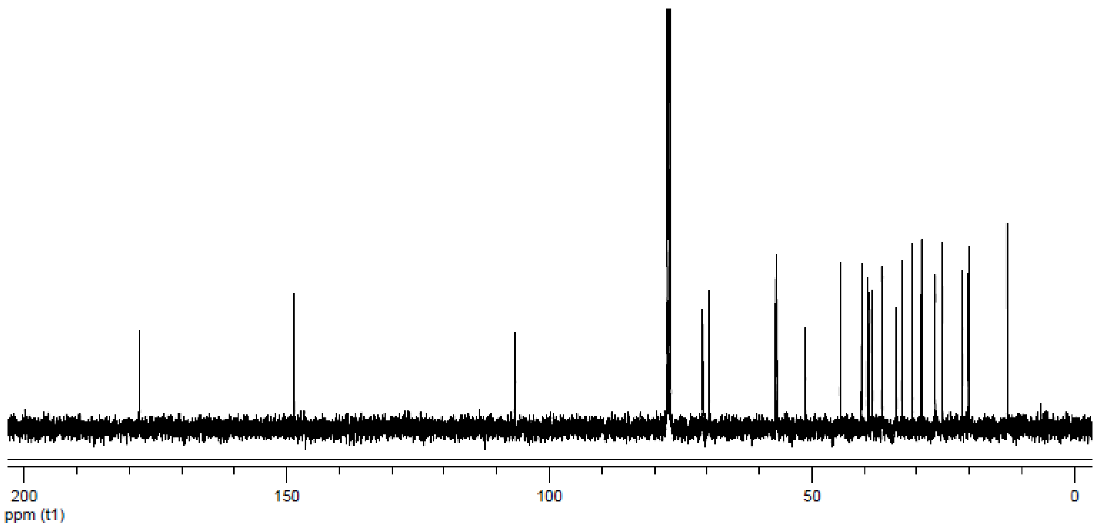

Figure S8. HMBC of Compound **9**.

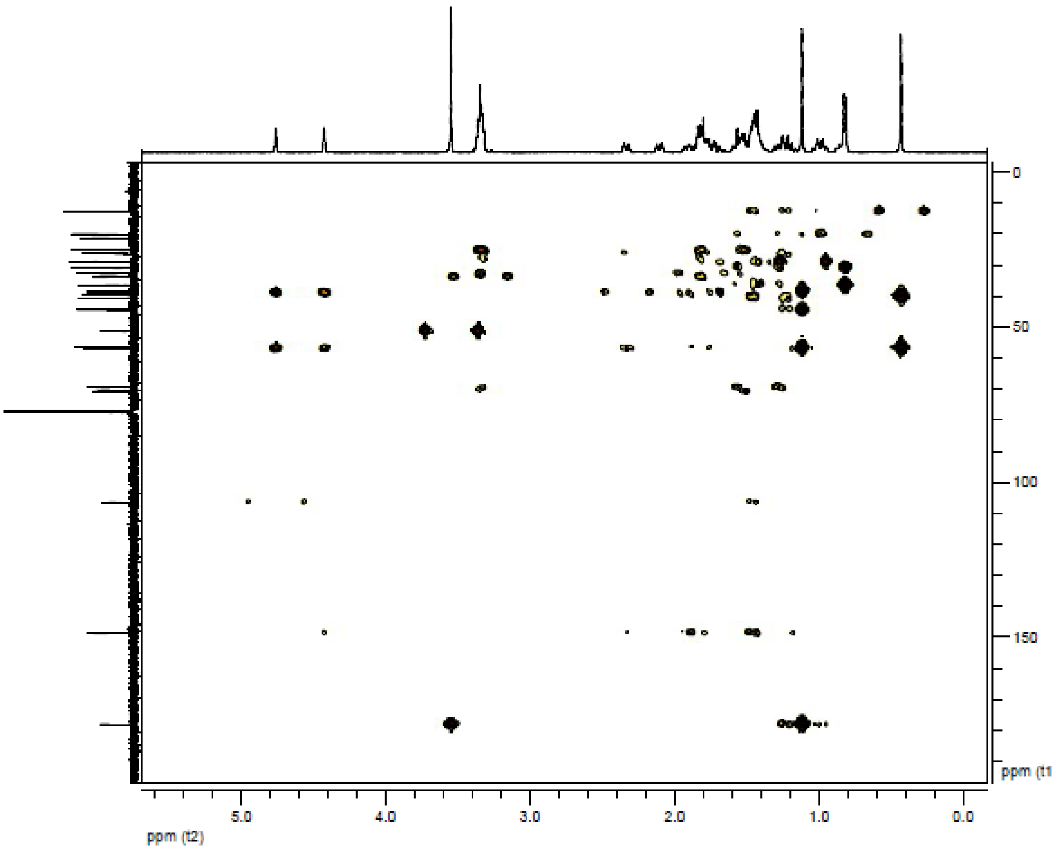

Figure S9. ^{1}H-NMR of Compound **11**.

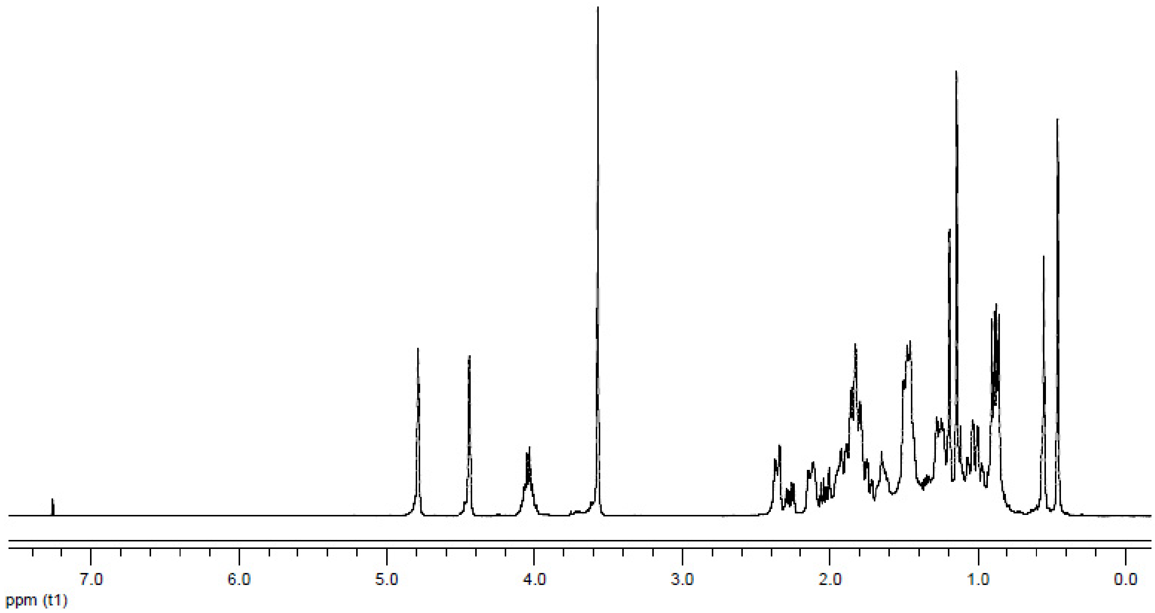

Figure S10. ^{13}C-NMR of Compound **11**.

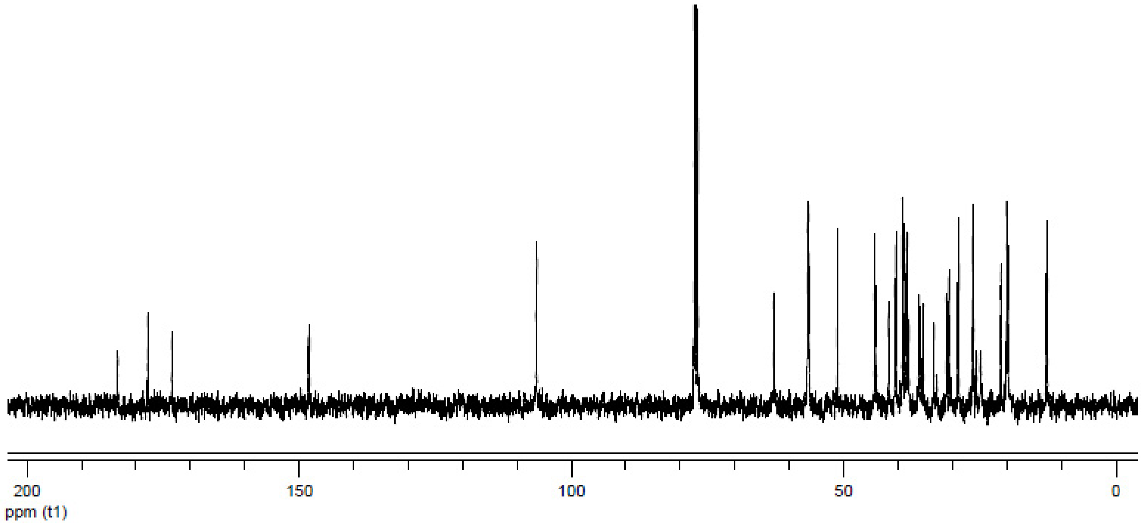

Figure S11. ^{1}H-NMR of Compound **15**.

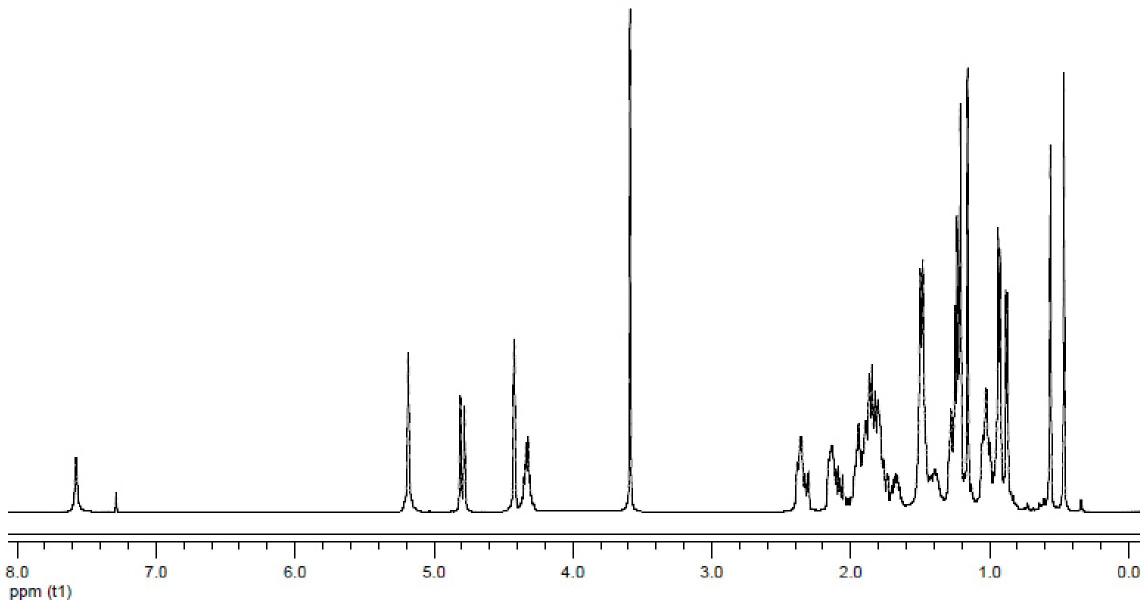

Figure S12. ^{13}C-NMR of Compound **15**.

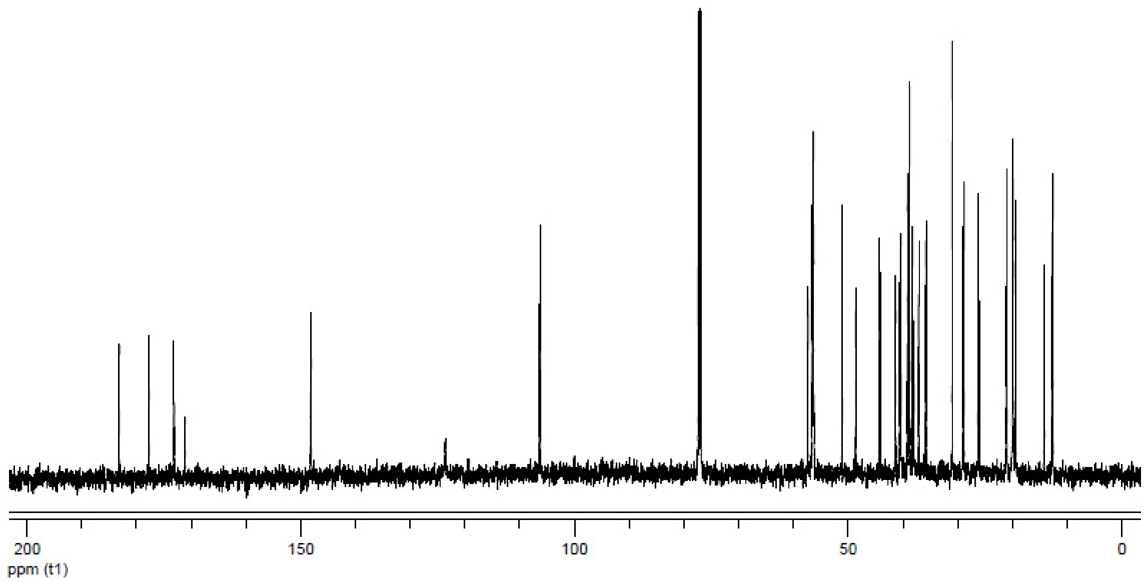

Figure S13. HMBC of Compound **15**.

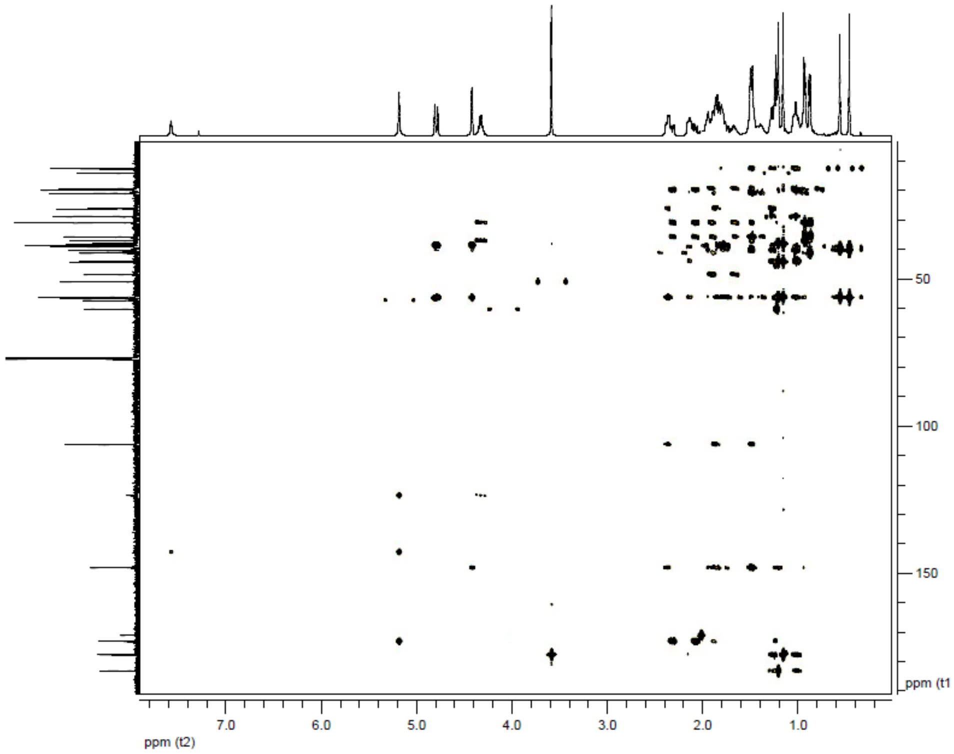

Figure S14. COSY of Compound **15**.

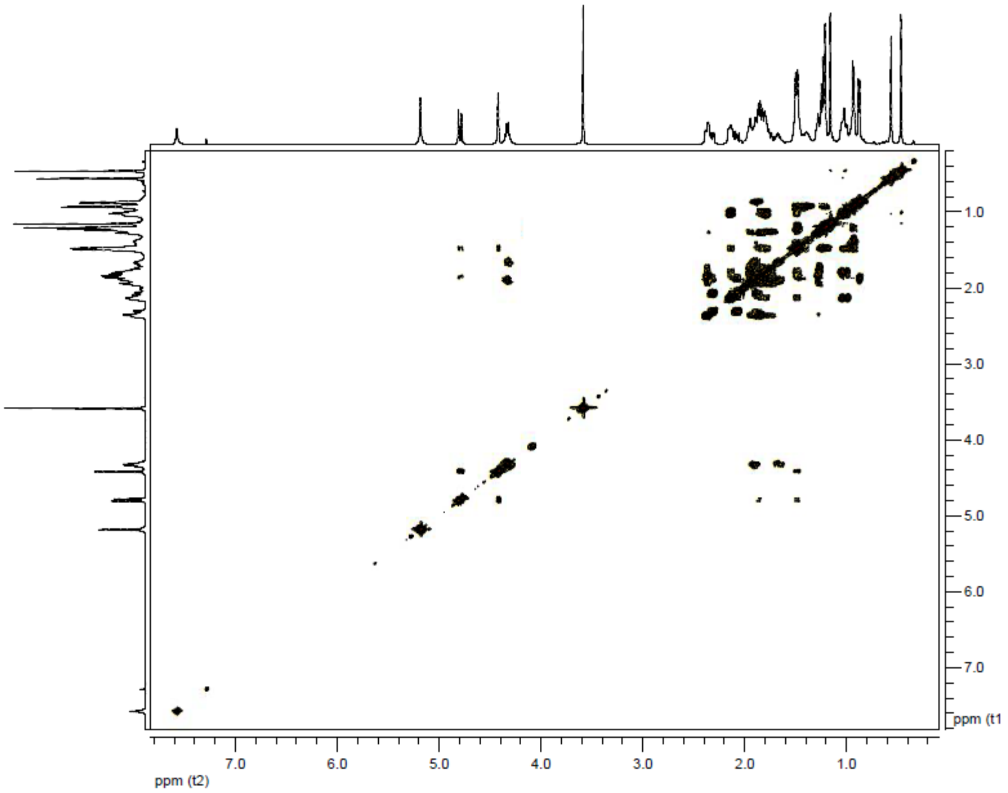

Supplement: Supplementary file 1 [file molecules-18-05936-s001.pdf]
